# Evolution of HLA-F and its orthologues in primate species: a complex tale of conservation, diversification and inactivation

**DOI:** 10.1007/s00251-020-01187-1

**Published:** 2020-11-12

**Authors:** N. Otting, N. G. de Groot, R. E. Bontrop

**Affiliations:** 1 Department of Comparative Genetics and Refinement, Biomedical Primate Research Centre, Rijswijk, The Netherlands; 2 IPD-MHC Database Nonhuman Primate Nomenclature Committee, Rijswijk, The Netherlands; 3 Department of Biology, Theoretical Biology and Bioinformatics, Utrecht University, Utrecht, The Netherlands

**Keywords:** HLA-F, MHC, Comparative genetics, Nonhuman primates

## Abstract

HLA-F represents one of the nonclassical MHC class I molecules in humans. Its main characteristics involve low levels of polymorphism in combination with a restricted tissue distribution. This signals that the gene product executes a specialised function, which, however, is still poorly understood. Relatively little is known about the evolutionary equivalents of this gene in nonhuman primates, especially with regard to population data. Here we report a comparative genetic analysis of the orthologous genes of HLA-F in various great ape, Old World monkey (OWM), and New World monkey (NWM) species. HLA-F-related transcripts were found in all subjects studied. Low levels of polymorphism were encountered, although the length of the predicted gene products may vary. In most species, one or two transcripts were discovered, indicating the presence of only one active F-like gene per chromosome. An exception was provided by a New World monkey species, namely, the common marmoset. In this species, the gene has been subject to duplication, giving rise to up to six F-like transcripts per animal. In humans, great apes, and OWM, and probably the majority of the NWM species, the evolutionary equivalents of the HLA-F gene experienced purifying selection. In the marmoset, however, the gene was initially duplicated, but the expansion was subjected afterwards to various mechanisms of genetic inactivation, as evidenced by the presence of pseudogenes and an array of genetic artefacts in a section of the transcripts.

## Introduction

The classical HLA-A, -B, and -C molecules are highly polymorphic and are usually expressed on the cell surface of nucleated cells. They play an important role in the initiation and regulation of adaptive immune responses, by presenting peptides derived from intracellular pathogens to receptors on various types of T cells. Some of the polymorphic epitopes, born on classical MHC class I molecules, are recognised by ligands on natural killer cells (Parham, Guethlein 2018). The non-classical MHC class I molecules in humans are designated HLA-E, -F, and -G and are characterised by low levels of polymorphism. These gene products display a restricted tissue distribution, and generally, but not exclusively, interact with receptors of the innate immune system; for example, HLA-E is a monitor for the manipulation of MHC class I expression by pathogens. In this particular example, the ligands are the CD94/NKG2 receptors, and interaction may augment or inhibit NK cell-mediated cytotoxicity and cytokine production (Lee et al. 1998). HLA-G is expressed on trophoblast cells of the placenta and is considered to function as a tolerogenic immunoregulator during pregnancy (Shiroishi et al. 2006; Persson et al. 2017).

The focus of this communication is on HLA-F, and in particular on its orthologous structures in nonhuman primates. Although the HLA-F gene was first described in 1990 (Geraghty et al. 1990), its precise functional significance still needs to be resolved and understood. Like the classical class I genes, the HLA-F gene contains 8 exons. In contrast, the messenger RNA of HLA-F lacks exon 7, and after translation this modification results in a shorter cytoplasmic tail. The valine residue at the end of the cytoplasmic tail plays a crucial role in the dislocation of HLA-F from the endoplasmic reticulum (Boyle et al. 2006).

The HLA-F protein is intracellularly expressed in resting lymphocytes such as B, T, NK, and monocytes (Wainwright et al. 2000). The cell-surface expression of HLA-F, however, appears to be a marker for an activated immune response. HLA-F can be encountered as an open conformer (OC), devoid of peptide, and Beta-2 microglobulin (ß2M), and in this context it may act as a ligand for inhibiting killer-cell immunoglobulin-like receptors (KIR) 3DL1 and 3DL2 (Goodridge et al. 2013). Moreover, HLA-F OCs may interact with the activating KIR3DS1, a receptor that is known to influence the outcome of various diseases, including HIV-1 disease progression (Burian et al. 2016; Garcia-Beltran et al. 2016; Kiani et al. 2018). The interaction with another activating NK cell receptor, KIR2DS4, is still a matter of debate (Persson et al. 2019).

It took considerable time to ascertain that HLA-F has the capacity to bind and present antigen segments. Elucidation of the crystal structure revealed an open-ended antigen binding groove that enables the binding of relatively long peptides (Dulberger et al. 2017; D'Souza et al. 2019). The open-ended groove likely results from an amino acid replacement of arginine (R) by tryptophan (W) at position 62 (R62W) in the peptide binding region. This mutation is only reported for the human and orangutan gene products and is not encountered in genetically closely related species such as chimpanzee and gorilla. Complexes of HLA-F/peptide/ß2M on the cell surface can only be recognised by the inhibitory immunoglobulin-like transcript receptors LILRB1 (also denoted as LIR1 or ILT2) and LILRB2 (LIR2 or ILT4) (Lepin et al. 2000; Dulberger et al. 2017; D'Souza et al. 2019). On the one hand, HLA-F expression on extra-villous trophoblast cells in the placenta and in pre-implantation endometrium hints towards a protective role in pregnancy (Hackmon et al. 2017; Persson et al. 2019). On the other hand, HLA-F polymorphism may play a role in the outcome of infectious diseases and in autoimmune disorders (Laaribi et al. 2018; Santos et al. 2018).

By definition, orthologues genes are related to each other by descent from a common ancestor. Orthologues of the HLA-F gene were identified at the transcription level in great apes (Adams, Parham 2001; Moscoso et al. 2007), rhesus macaques (Otting, Bontrop 1993), sooty mangabeys (Heimbruch et al. 2015), and cotton-top tamarins (Watkins et al. 1990). These sequences often represent partial transcripts. As mentioned earlier, a feature of nonclassical HLA genes involves its low levels of polymorphism. At present, 44 HLA-F alleles archived in the Immuno *Polymorphism* Database (IPD-IMGT/HLA release 3.40.0) are translated in only six allotypes (Robinson et al. 2020). Our goal was to investigate the presence of the evolutionary equivalents of HLA-F and to determine levels of allelic polymorphism in cohorts of nonhuman primate species.

## Materials and methods

### Animals and samples

The panel consisted of samples of great ape species, namely, 26 Western chimpanzees (*Pan troglodytes verus, Patr*), 5 Lowland gorillas (*Gorilla gorilla, Gogo*), 3 Bornean (*Pongo pygmaeus, Popy*), and 3 Sumatran (*Pongo abelii, Poab*) orang-utans; samples of Old World monkeys, namely, 33 Indian rhesus macaques and 27 Chinese rhesus macaques (*Macaca mulatta, Mamu*), 101 Indochinese cynomolgus macaques (*Macaca fascicularis, Mafa*), 26 Southern pig-tailed macaques (*Macaca nemestrina, Mane*), and 11 olive baboons (*Papio anubis, Paan*); and samples of New World monkeys, namely, 31 marmosets (*Callithrix jacchus, Caja*), 10 grey-bellied night-monkeys (*Aotus lemurinus, Aole*), and 13 cotton-top tamarins (*Saguinus oedipus, Saoe*). All groups, except for the orangutans and marmosets, included samples of related (parent/child) animals, allowing the segregation of polymorphic genes to be studied.

For the investigation of MHC-F polymorphism in nonhuman primates, frozen cells or RNA samples were obtained from different sources. EBV-transformed B cells are present of Western chimpanzees that had been bred for decades at the BPRC. Moreover, EBV-transformed B cells of six orang-utans and one Lowland gorilla are available in our tissue bank, whereas we obtained peripheral blood mononuclear cells (PBMCs) of four additional Lowland gorillas during the course of the study. RNA samples from various macaque species were still available from an earlier study (Otting et al. 2017). Furthermore, samples of olive baboons, that were characterised for their classical MHC class I repertoire, were still present (van der Wiel et al. 2018). Immortalised B cells and PBMCs of marmosets that are housed at the BPRC facilities were used. Furthermore, the BPRC has in its tissue bank frozen B cells of grey-bellied night monkeys and cotton-top tamarins.

### RNA isolation, cDNA synthesis, and amplification

RNA was isolated from thawed immortalised B cells or PBMCs using the RNeasy kit (Qiagen, Valencia, Ca, USA). First-strand cDNA syntheses were performed on the RNA samples with the RevertAid-kit cDNA synthesis kit as recommended by the supplier (Thermo Fisher Scientific, Waltman, Ma, USA). For the design of primers that amplify the full-length MHC-F gene, genomic data on the chimpanzees (Adams, Parham 2001; Shiina et al. 2006; Gleimer et al. 2011), the rhesus macaques (Daza-Vamenta et al. 2004), and the marmosets (Kono et al. 2014) was used. To avoid the co-amplification of other class I genes, two sets of F-specific primers were devised, and nested PCR’s were performed on all samples (Table 1). The initial PCR’s on cDNA were performed using Phusion Hot Start II DNA polymerase (Thermo Fisher Scientific) in 50 μl mixtures with the primer-sets F2/R2. The amplification protocol consisted of an initial step of 30 s at 98 °C, followed by 20 cycles of 98 °C for 10 s, 55 °C for 15 s, and 72 °C for 30 s, and the last extension step was extended to 1 min. Of these amplifications, 5 μl was used for a nested PCR with the primer-sets F1/R1, under the same conditions as mentioned above. The nested PCR products were run on a 1% agarose gel, excised from this gel, and purified using the GeneJet gel extraction kit (Thermo Fisher scientific).

**Table 1:** PCR primers used in this study

	Primer ID	Sequence 5′—3'
Great apes	Patr-F-F1	CCCACGCACCCCGCGGGACTC
	Patr-F-F2	GAAGCCAATCAGTGTCGCAG
	Patr-F-R1	GATATCTTGCTTCTCAGTCCC
	Patr-F-R2	GGCACAAGTGCAATTCTGCTAC
OWM	Mamu-F-F1	CTCAGATTCTCCCCAGACGCG
	Mamu-F-F2	CACTCCCATTGTGTGCGGAG
	Mamu-F-R1	GGGGTGAAGACAYATTTGGAC
	Mamu-F-R2	GGACATGGGGGTAGGCTGG
NWM	Caja-F-F1	CTAAAGTCCCCACGCACCCACGG
	Caja-F-F2	GCCAATCAGCGTCGCCGT
	Caja-F-R1	CAGGGAKGAAGACRCATTTGGAC
	Caja-F-R2	GACATGGGGGTGGACTGGTCC

### Sanger sequencing and cloning

Direct sequencing reactions on the PCR products were performed in the forward and reverse directions, using the BigDye terminator cycle sequencing kit (Thermo Fisher scientific). For priming of the sequencing reaction, the F1 and R1 primers that amplified the respective samples were used (Table 1). The samples were run on a Genetic Analyzer 3500 capillary system. The resulting peak patterns were analysed with MacVector™ software, version 16.0.10 (Cambridge, UK). Direct sequencing of the MHC-F PCR products on the Genetic Analyser 3500 often led to peak patterns that display double peaks at particular positions because of heterozygosis. First the homozygous samples were analysed, followed by a comparison of peak-patterns in related animals that shared one allele. With this strategy it was possible to determine unambiguously the full-length alleles in the majority of animals. In cases where the alleles could not be identified, a cloning step was introduced. These PCR products were then ligated using the CloneJet PCR cloning system (ThermoFisher Scientific). After transformation into XL1-blue bacteria (Stratagene, La Jolla, California), 12 colonies were selected for culturing and plasmid isolation. As described above, sequencing of the plasmid DNA was performed, using the forward and reverse primers provided in the CloneJet system.

All unreported MHC-F alleles, based on at least two independent PCR reactions, were submitted to the European Nucleotide Archive (ENA) (www.ebi.ac.uk/ena) as well as to the nonhuman primate section of the IPD database (Maccari et al. 2017).

### Next-generation sequencing

Cloning and sequencing illustrated that more than two MHC-F genes are present in marmosets. To obtain all F-sequences present per animal, next-generation sequencing (NGS) on a PacBio platform was performed. The Caja-F1/R1 primers (Table 1) were tagged with 16 nucleotide barcodes to identify the samples within a pool. With these tags nested PCRs, electrophoresis, and purification were performed as indicated above. The DNA concentrations of the PCR-samples were measured using the Qubit dsDNA HS assay kit and Qubit 2.0 Fluorometer (Thermo Fisher Scientific). The samples were pooled with equal quantities of DNA, and the pools were purified twice using AMPure XP beads (Beckman Coulter, Woerden, The Netherlands) at a 1:1 bead to DNA volume ratio. The DNA concentrations of the purified pooled samples were measured using the Qubit dsDNA HS assay kit and Qubit 2.0 Fluorometer (> 2.5 μg total DNA per pool). SMRTbell library generation and sequencing were performed by the Leiden Genome Technology Center using a PacBio Sequel instrument with P6-C4 sequencing chemistry. The data were demultiplexed based on the unique barcoding, and the individual samples were analysed using Geneious Prime software version 2019.1.3 (Biomatters Ltd, New Zealand). The sequence reads were mapped to a library of *Caja-F* sequences, found earlier by cloning and sequencing. Unused reads were de novo assembled, and trimmed for the Caja-F1/R1 primers. New sequences that were confirmed in two separate PCR samples were submitted to the above-mentioned databases.

### Phylogenetic analyses, nomenclature, and calculation of d*N*/d*S* ratio

Neighbor-Joining trees were constructed with the MEGA 7.0.18 program (Kumar et al. 2016). The evolutionary distances were computed using the Maximum Composite Likelihood method, and bootstrap values were calculated based on 1000 replicated.

**Fig. 1:**
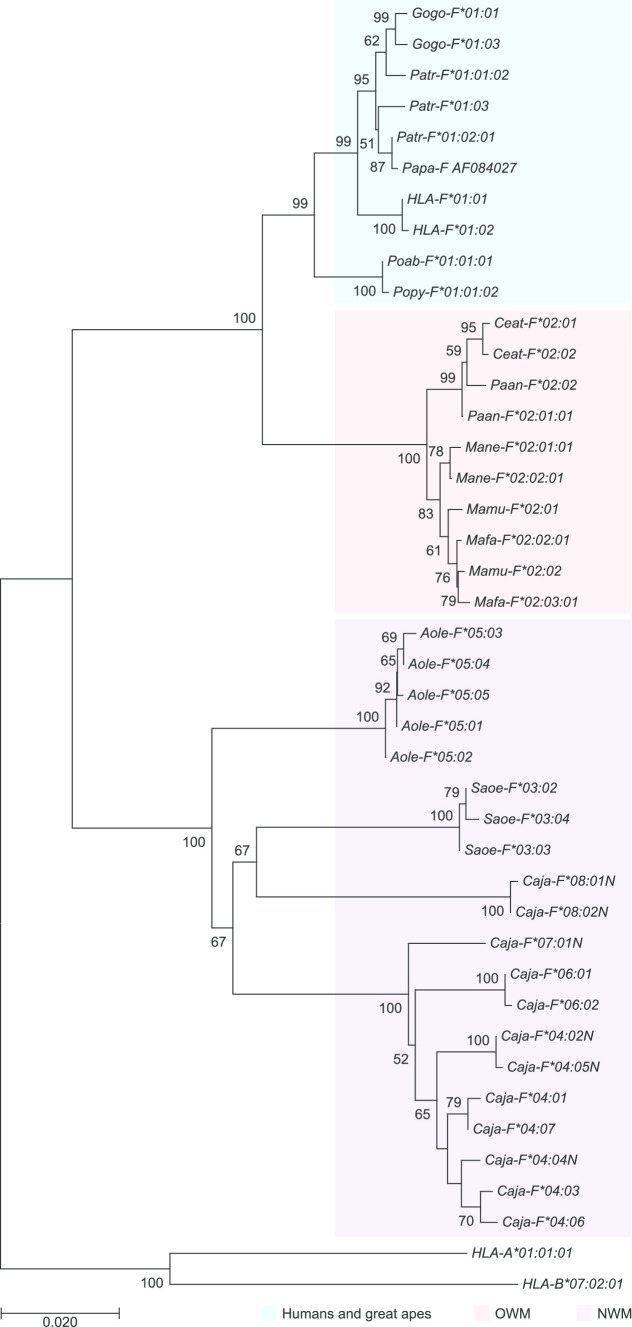
Phylogenetic tree of *HLA-F* and representatives of nonhuman primate *MHC-F* lineages. *HLA-A* and *-B* sequences are used as outgroup. The Bonobo (*Pan paniscus*, *Papa-F*) sequence is retrieved from Genbank and the accessionnumber is indicated, whereas the alleles of the sooty mangabey (*Cercocebus atys, Ceat-F*) were downloaded from the IPD database (www.ebi.ac.uk/ipd/mhc/). Relevant bootstrap values are indicated

Sequence alignments and phylogenetic analyses were used to investigate the grouping of nonhuman primate alleles and to designate them according to standardised nomenclature rules (de Groot et al. 2019). The great ape alleles cluster with *HLA-F* and have received the lineage number F*01 in their designations (Fig. 1). A lineage is defined as a group of similar alleles that cluster together in phylogenetic analyses and are considered to originate from an ancestral allele. Cynomolgus and rhesus macaque sequences were already archived as Mafa-F*02 and Mamu-F*02 in the nonhuman primate section of the IPD database. The alleles of the pigtailed macaque, and the olive baboon group into the F*02 branch, have been given designations accordingly. For cotton-top tamarins and common marmosets, the lineages Saoe-F*03 and the Caja-F*04, respectively, were already designated in the IPD database. The night monkey alleles were given the lineage number F*05 in their names, whereas additional lineages in the marmoset were designated *06, *07, and *08.

The number of nonsynonymous substitutions per nonsynonymous site (dN) and the number of synonymous substitutions per synonymous site (dS) between sequences were calculated with the MEGA 7.0.18 program using the Nei-Gojobori method (Jukes-Cantor p-distance correction for multiple substitutions at the same site) (Nei, Gojobori 1986). This was performed for complete exons 2–3, and the remaining exons 1–4–5–6–8 of the *MHC-F* gene for cynomolgus macaque (*Mafa-F*), rhesus macaque (*Mamu-F*), and pigtailed macaque (*Mane-F*). The OWM *MHC-F* sequences contain an insert (6 nucleotides) in exon 2 as compared with the *MHC-F* sequences in humans and great apes (Suppl. Fig. 1). As a result, it is difficult to assign which triplets in the sequences encode for the contact residues in these OWM species, also because the crystal structure of these molecules is lacking. Therefore, we have not calculated the d*N* and d*S* for the antigen recognition site (ARS) and the non-ARS for the *MHC-F* sequences of the indicated macaque species. The SE was calculated using a bootstrap of 1000 replicates.

## Results

### Allele discovery

In total, 98 *MHC-F* alleles were detected in this study, and they are listed with their ENA accession numbers and the names of reference animals (Suppl. Table 1). In cases where alleles confirmed sequences that had already been catalogued in the nonhuman primate part of the IPD database (https://www.ebi.ac.uk/ipd/mhc/group/NHP/), this has been indicated. A full-length DNA alignment of the alleles, representing all the investigated species, is depicted (Suppl. Fig. 1). Moreover, a separate alignment of rhesus and cynomolgus macaque sequences is provided (Suppl. Fig. 2). Additional alignments of nonhuman primate *MHC-F* alleles can readily be created and viewed, in DNA and in protein, on the IPD website. The sequences, grouped per gene and primate species, are downloadable in Fasta format. All sequences described in this communication lack exon 7 in the mRNA transcripts and are therefore considered to represent orthologues of *HLA-F*.

**Fig. 2:**
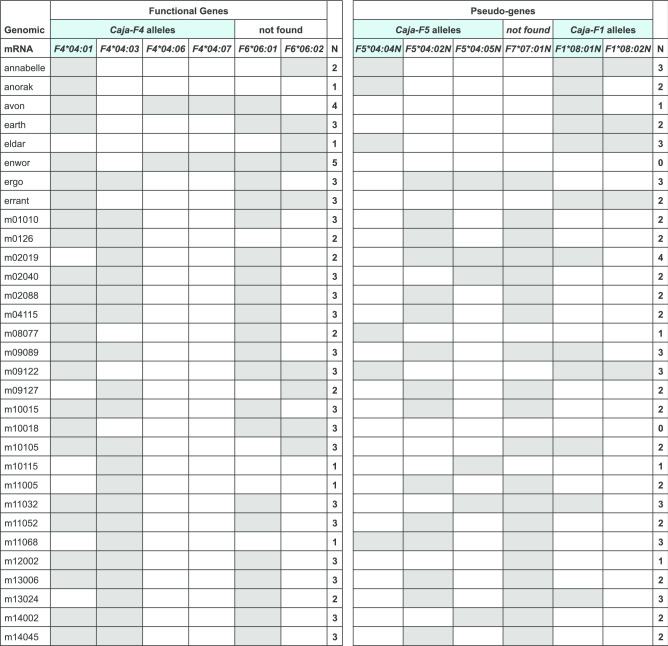
The *Caja-F* alleles present in the 31 common marmosets. The genes, found during analyses on the G/F region, are shown in the upper row. Transcripts that are identical to genomic sequences are depicted in blue. Alleles that translate into functional proteins are listed in the *left-hand* panel. Alleles of pseudogenes have an N in their designations and are listed in the *right-hand* panel. The number of alleles per animal is listed

*Great apes*: In the Western chimpanzee population analysed, five *Patr-F* alleles were discovered, giving rise to three Patr-F allotypes (Suppl. Table 1). The PCR primers (Patr-F/R) that were designed on the basis of chimpanzee genomic data turned out to be applicable for amplification of the *MHC-F* in gorillas. In the Lowland gorilla, two *Gogo-F* alleles representing two allotypes were detected. The primer set was also used to analyse the orangutan samples. However, a subsequent cloning and sequencing step were needed for these samples, as the majority of clones appeared to represent *MHC-A* and *-B* gene transcripts. Nevertheless, one *Popy-F* and two *Poab-F* alleles were discovered in the Bornean and the Sumatran animals, respectively. Overall, only one or two alleles were detected per animal, suggesting that for these three great ape species one *F* gene is present per haplotype. On average, the gene transcripts in great apes are similar to *HLA-F*, suggesting a high level of conservation (Suppl. Fig. 1). The similarity is reflected in the allele designations, and all great ape alleles were given lineage number F*01 (Fig. 1). Our studies confirm that the amino acid replacement at position 62, arginine (R) by tryptophan (W), encoded by TGG, is shared only by humans and orangutans (Suppl. Fig. 1, exon 2). Comparison with the OWM and NWM lineages illustrates that the arginine (R) at this position appears to be ancestral and that the replacement by the tryptophan residue most likely happened independently in the human and orangutan lineage and thereafter became fixed in these two species.

*Old World monkeys*: In our panel of rhesus macaques, 22 alleles were discovered. We confirmed a *Mamu-F* sequence, reported more than two decades ago (Otting, Bontrop 1993), and is now designated Mamu-F*02:04:01. The PCR primer-sets designed for the rhesus macaque (Mamu-F/R) also amplified the corresponding gene in cynomolgus- and pigtailed macaques. This led to the discovery of 31 *Mafa-F* and 11 *Mane-F* alleles, respectively. Translation of the transcripts resulted in the establishment of 16 Mamu-F, 20 Mafa-F, and 7 Mane-F allotypes. Comparative analysis indicates that exon 3, encoding the alpha 2-domain that forms the scaffolding of the antigen binding cleft, is vastly conserved in humans, great apes, and OWM (Suppl. Fig. 1). Although the number of alleles/allotypes encountered in the populations of macaques is relatively high, the differences between them are minimal (Suppl. Fig. 2). The point mutations are not restricted to exon 2 but are scattered over exons 1 up to 5. This suggests that in macaque species a purifying type of selection is operative, which is in line with the observation that some alleles are shared by two species of macaque (Suppl. Table 1). The ratios for dN/dS for complete exons 2–3 and the remaining exons 1–4–5–6–8 were found to be < 1 for the three different macaque species (Table 2), which is indicative for purifying selection. However, some caution is required by the interpretation of these ratios, as the data set comprises of a list of sequences, and does not signify the diversity in a population where the relevant frequency levels are known. In the olive baboons, four different *Paan-F* alleles were observed, representing three allotypes. The *MHC-F* sequences of OWM are much alike, and all have been given the lineage number F*02 in their allele names. As in humans and great apes, in all animals of the OWM species, only one or two alleles were detected, confirming the presence of one gene per chromosome.

**Table 2:** The average number of dN and dS for the cynomolgus (Mafa), rhesus (Mamu), and pigtailed (Mane) macaque *MHC-F* gene separately calculated for the complete exons 2–3 and remaining exons. In brackets the SE is given. N represents the number of codons that are compared between the different numbers of alleles for the indicated species

	Complete exons 2–3		
	Mafa-F (*N* = 184)	Mamu-F (*N* = 184)	Mane-F (*N* = 184)
dN (SE) dS (SE)	0.002 (0.001) 0.014 (0.006)	0.003 (0.001) 0.013 (0.006)	0.001 (0.001) 0.010 (0.004)
dN/dS	0.14	0.23	0.1
	Remaining exons (exons 1-4-5-6-8)		
	Mafa-F (*N* = 165)	Mamu-F (*N* = 165)	Mane-F (*N* = 165)
dN (SE) dS (SE)	0.005 (0.002) 0.017 (0.007)	0.003 (0.002) 0.008 (0.003)	0.005 (0.002) 0.014 (0.006)
dN/dS	0.29	0.38	0.36

*New World monkeys*: The primers designed for common marmosets turned out to be effective as well for the amplification of *F-*genes in two other cohorts of NWM: the grey-bellied night monkeys (*Aole-F*) and the cotton-top tamarins (*Saoe-F*). In these animals, five and three alleles/allotypes were detected, respectively. As observed in the OWM and great apes, only one or two alleles were present per animal. The *MHC-F* alleles of night monkeys and cotton-top tamarins differ profoundly and have been given distinctive lineage numbers F*05 and F*03, respectively (Fig. 1; Suppl. Fig. 1).

In common marmosets, 854 kb of the MHC region that corresponds to the HLA-A/G/F segment was sequenced and annotated (Kono et al. 2014). The results of this study illustrate segmental duplications, leading to the description of at least five F-like paralogues, which were numbered accordingly *Caja-F1* up to *-F5*, based on the order on the chromosome. Only *Caja-F4* represents a functional gene, and the coding sequence (CDS) was named and archived as Caja-F*04:01 in the IPD database. Paralogous genes have the tendency to accumulate genetic variation, and therefore such genes encode comparable proteins, often with similar but not identical functions. Sanger sequencing of the marmoset *MHC-F* amplicons revealed indeed a mixture of several transcripts. For the sake of completeness, next-generation sequencing on a PacBio platform was performed on the samples of the common marmoset. These analyses yielded up to six *Caja-F* sequences per animal. In total, twelve distinct *Caja-F* transcripts were detected in 31 marmosets, of which only six may give rise to a functional protein (Fig. 2). The other six are characterised by early stop codons and have been accorded an N in the allele names to indicate that they are not translated in functional proteins.

### Deciphering orthologous and paralogous relationships of F genes in common marmosets

**Fig. 3:**
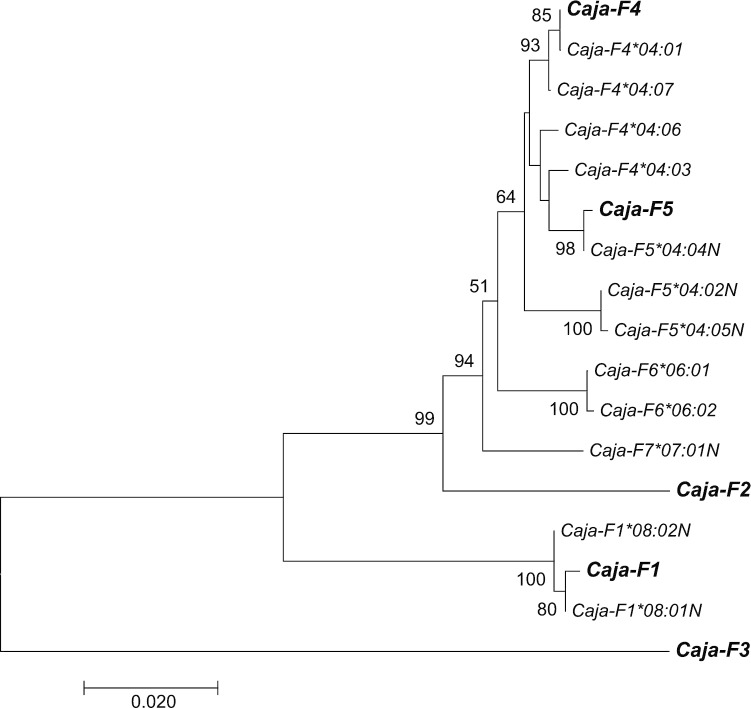
Phylogenetic tree of *Caja-F* alleles based on mRNA/cDNA sequencing and on *Caja-F* genes found with genomic sequencing of the G/F region (denoted as Caja-F1, -F2, -F3, -F4 and -F5). Relevant bootstrap values are indicated

To sort out the relationship of the marmoset sequences based on mRNA/cDNA sequencing and the genomic sequences *Caja-F1* up to *-F5* (Kono et al. 2014), the coding sequences (CDS) of latter were withdrawn from the IPD database, and phylogenetic analyses were performed (Fig. 3). As can be observed, the CDS of *Caja-F1* gene clusters together with our F*08 transcripts. The allele F*08:01N appears to be identical to the *Caja-F1* CDS, except for a 38-nucleotides-long deletion in exon 3 that is present in all the transcripts in our panel. Therefore, the allele names were extended with the gene number to become Caja-F1*08:01N and Caja-F1*08:02N. Both alleles differ in length from the other *Caja-F* orthologues, and a stop codon is present in exon 4, confirming the claim that *Caja-F1* is a pseudogene (Fig. 2).

Transcripts of the *Caja-F2* and *-F3* pseudogenes were not detected in the present study, which could be due to a primer misfit.

The Caja-F*04:01 allele, which translates into a functional protein, is detected in the majority of animals and is identical to the CDS of the *Caja-F4* gene (Fig. 3). This gene is functional and seems to control a small number of alleles, the names of which are also extended with the gene number *F4*. Caja-F4*04:03 may be an allele of the same gene, present on the other chromosome*.* Particularly striking is the number of reads of this allele in NGS, which is often tenfold higher than for Caja-F4*04:01 (data not shown). This suggests that alleles of the same *F* orthologue may display substantial heterogeneity with regard to its levels of transcription and expression. The other two candidates that are likely encoded by *Caja-F4* are *04:06 and *04:07, which are linked but rare in the panel. It is conceivable that these two transcripts are encoded not by one but by two genes, suggesting another duplication. At this stage, however, we are hesitant to introduce another gene number, as this is not supported by the available genomic data.

The DNA alignment and phylogenetic tree showed that the F*04:04N transcript is identical to the CDS of the *Caja-F5* pseudogene, except for a 63-nucleotides-long deletion in exon 3 (Fig. 3; Suppl. Figure 1). The Caja-F*04:02N and -F*04:05N transcripts are probably alleles of *Caja-F5* but have a 2-nucleotide-long deletion in exon 3, causing a frameshift in the translation. Subsequently, the gene names of these non-functional alleles have been extended to Caja-F5*04:02N, -F5*04:04N, and -F5*04:05N. The alleles of *Caja-F4* and the pseudogene *Caja-F5* are similar, and cluster together in the phylogenetic tree. It is possible that *Caja-F5* is the result of a relatively recent duplication of the *Caja-F4* gene.

The Caja-F*06:01 and F*06:02 transcripts are doubtless two alleles of the same gene, and we have encountered individuals that are homozygous for this entity. These transcripts do not match with any of the reported genes *Caja-F1* up to *-F5* and are probably encoded by an unknown orthologue. Indeed, the published genomic sequence of the MHC class I G/F segment features a gap (Kono et al. 2014). It is possible that a sixth *F-*like gene maps in this missing area, and the two transcripts have been named Caja-F6*06:01 and -F6*06:02, respectively. In Caja-F*07:01N a single nucleotide insertion in exon 4 results into a frameshift and early stop. The coding gene for this allele is also unknown, and we have numbered the gene *Caja-F7*.

The sequencing analyses have shown that two functional *F* genes, *Caja-F4* and *-F6*, may be present per chromosome (Fig. 2). Since pedigree data is lacking, it is not possible to assign the alleles to the maternal or paternal chromosomes or to define *Caja-F* haplotypes. Moreover, the NWM family of *Callitrichidae*—that includes both marmosets and tamarins—are known to produce bone marrow chimeric twins (Sweeney et al. 2012). This implies that blood and other cells from the hematopoietic lineage are shared by twins, which may complicate MHC haplotyping in these animals. Analyses of the cotton-top tamarins, another member of the Callitrichidae family, however, showed that only one or two *Saoe-F* alleles per animal were present.

### The length of exon 2 and exon 5 in MHC-F shows variation among nonhuman primate species

**Fig. 4:**
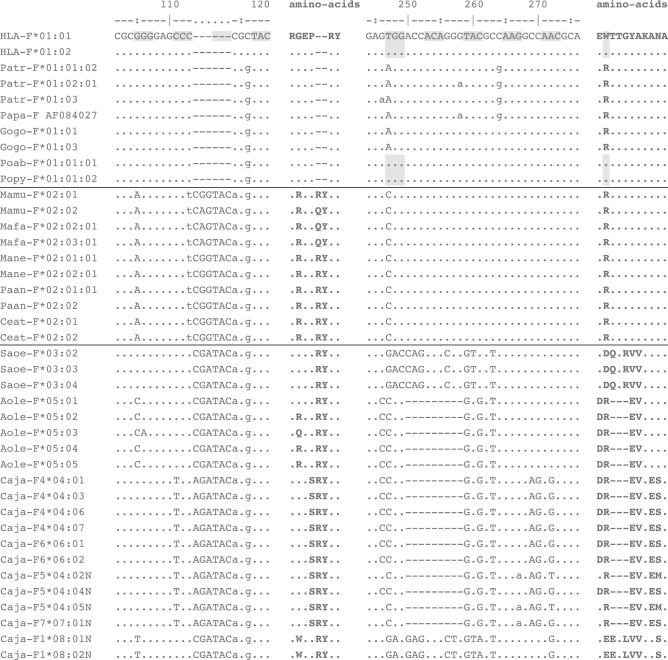
DNA alignment of two segments of exon 2, with translations in amino acids. Representative alleles of all nonhuman primate species are present. *Lowercase* letters refer to synonymous differences as compared with the HLA-F*01:01, whereas *capitals* indicate nonsynonymous changes. The *hyphens* represent missing nucleotides in comparison to the human reference sequence. The codon for tryptophan in human and orangutan sequences at position 247-249 is *shaded*. The alignment of the full-length sequences is included as Supplementary Fig. 1

All OWM and NWM *MHC-F* alleles detected in this study share an insertion of six nucleotides in exon 2, which results in the introduction of two amino acids, namely, an arginine (R) or glutamine (Q) by the first codon, and tyrosine (Y) by the second (Fig. 4, left-hand panel). This modification is absent in the orthologous *F* sequences encountered in humans and great apes. The *HLA-A* and *-B* genes, and the orthologous *MHC-A* and *-B* genes in all primate species studied thus far, have a 270-nucleotides-long exon 2. The insertion in OWM *MHC-F* genes extends the length of exon 2 to 276 nucleotides. Therefore, the most parsimonious explanation is that the insertion in the *F* gene took place early in the OWM/NWM lineage, but subsequent to its radiation from the human/hominoid lineage. It is anticipated that the two amino acid extensions of the alpha 1 domain in Old and New world monkey F allotypes have an impact on the composition of the peptide binding site, and as such affect the repertoire of peptides that can be bound. The extent to which this might be the case is at present not understood, however, and needs further investigation.

The night monkey and marmoset genes share a 9-nucleotides-long deletion in exon 2 that encodes an essential part of the peptide binding site (Fig. 4, right-hand panel). In protein, the deletion is situated next to the R62W replacement that converted the composition of the peptide binding site of the F protein in humans and orangutans (Dulberger et al. 2017). The sharing of this modification by two distant NWM species suggests that it was generated in an ancestral event. In the cotton-top tamarin transcripts, however, this nucleotide deletion of nine base pairs is absent, and the data suggest an insertion of at least 9 nucleotides in this region of exon 2. The boundaries of this insertion may be even longer. By way of illustration, the GAC codon in the Saoe-F*03 lineage leads to an R62D (Arginine by Aspartic acid) replacement. In all other alleles of the great apes, and the OWM, a T/A/CGG codon is observed and is substantially different in composition. In fact, the entire amino acid motif DQTRVV (Fig. 4, right-hand panel), and its codon usage deviates from the other known F allotypes/alleles in primates that have been recorded up until now. This would be explained by a recombination with another gene that has donated a segment. The deletion, however, is not seen in the Caja-F1*08, which differs profoundly from the other *Caja-F* genes (Suppl. Fig. 1). An orthologous gene of *Caja-F1* in the tamarins could be the candidate donor gene. The GAG codon in this gene is not too distant but results in an R62E switch (Arginine to Glutamic acid). The Caja-F1*08 sequences show several signatures of inactivation, such as a 38-nucleotides-long deletion at the end of exon 3 (Suppl. Fig. 1), leading to a frameshift and an early stop during translation in a protein. It has been documented that sections of pseudogenes in the immune system are used as substrates to recruit sequence segments (Doxiadis et al. 2006). The main conclusion, however, is that during the evolution of *HLA-F* and its orthologues, the composition of the peptide binding groove in different species has been subjected to several independent molecular events.

The alignments of the *MHC-F* sequences revealed several peculiarities, and these genetic modifications took place during the evolution of NWM (Suppl. Fig. 1). First of all, as compared with humans and great apes, the differences in exon 3 in OWM are mostly synonymous. In the NWM, however, exon 3 has accumulated a high number of nonsynonymous point mutations in comparison to great apes and OWM. Nevertheless, after its diversification, the exon 3 sequence data suggest that in contemporary times purifying selection is operative on this section of the corresponding gene product in common marmosets (Suppl. Fig. 1). A similar feature, but to a lesser extent, is seen for the other exons.

All *F* genes in NWM share a four or five codon deletion in exon 5 (Suppl. Fig. 1). The grey-bellied night monkey has an additional 3-nucleotide-long deletion in this exon, six codons downstream. The *F* alleles in common marmosets and cotton-top tamarins feature extensions of three codons at the end of the exon, which more or less neutralises the length of the deletion. As this exon encodes the transmembrane section of the *F* gene product, grey-bellied night monkeys appear to have the smallest known transmembrane section of all primates.

### OWM and common marmosets: F-gene isoforms generated by alternative splicing

Direct sequencing of the PCR products obtained from macaques has often displayed a background profile, reflected by a low peak-pattern, starting directly after exon 1. This suggests that an insertion or deletion is present in a section of the transcripts. Subsequent cloning and sequencing of the samples revealed two anomalies that represent alternative spliced isoforms of the transcript. One isoform contained an insert of 130 nucleotides of intron 1, whereas the second isoform lacked the first 52 nucleotides of exon 2. Both isoforms result in a frame shift and the introduction of an early stop codon in exon 2. In three out of 11 baboons, the same isoforms were observed. The 52-nucleotide-long deletion was detected in two baboons, and one animal was positive for the insertion of intron 1. This illustrates that the generation of isoforms is shared between OWM species. In all the samples, these isoforms represent a minority as compared with the full-length transcripts. However, we cannot exclude that this phenomenon may occur more frequently in other tissues.

The cloning and Sanger sequencing revealed that in the common marmoset many transcripts had deletions or insertions. The purpose of the NGS step was twofold: to generate abundant reads to ensure that all *F* alleles per animal were detected and to inventory all the gaps and inclusions in the alleles. Some gaps were observed in only one animal, and these are considered to be PCR or sequencing errors. The deletions or insertions present in at least three animals, and that result from alternative splicing of mRNA, have been categorised (Table 3). Most often a deletion at the start of exon 2 or exon 3 was observed; for example, the 118-nucleotide-long deletion in exon 2 is present in all but the Caja-F1*08 sequences. In several cases, the complete exon 5 is missing, whereas in other alleles intron 1 is incorporated. This latter phenomenon is also seen in the *F* alleles in OWM.

**Table 3:** Deletions and insertions in *Caja-F* transcripts

			Present in:				
Position	Size (nt)	*Caja-F* chain	*Caja-F4*	*Caja-F5*	*Caja-F6*	*Caja-F7*	*Caja-F1*
Deletions							
61–64	4	Last part exon 1					*08:01N
65–125	61	First part exon 2	*04:03		*06:01	*07:01N	
65–182	118	First part exon 2	*04:01/03/06/07	*04:02N/04N/05N	*06:01/02	*07:01N	
330–392	63	2 nt exon 2—exon 3		*04:02N/05N			
331–399	69	1 nt exon 2—exon 3		*04:02N/05N			
332–370	39	First part exon 3				*07:01N	
332–394	63	First part exon 3	*04:01/03/06/07	*04:02N/04N/05N	*06:01/02	*07:01N	
332–405	74	First part exon 3	*04:01/03/07	*04:04N/05N	*06:01/02	*07:01N	*08:01N
579–616	38	Last part exon 3					*08:01N/02N
884–997	114	Whole exon 5	*04:01	*04:02N/05N		*07:01N	*08:01N
Inclusions							
	129	Intron 1		*04:04N		*07:01N	
	130	Intron 1					*08:01N
997	18	Behind exon 5	*04:01	*04:02N/05N	*06:02		

The positions and sizes are based on an alignment of only *Caja-F* sequences

## Discussion

Orthologues of the *HLA-F* gene are present in the great apes, OWM, and NWM. This illustrates that the *F* gene, which lacks exon 7, is old, and that a primordial structure was already present in a common ancestor that lived approximately 30 million years ago. The majority of the nonhuman primate species that was analysed, encodes one functional *F* gene per chromosome. On average, low levels of allelic polymorphism are encountered in each species, which would suggest a conserved function that has been remained over time. In this context, one should realise, however, that a single substitution may have a great biological impact. Single nucleotide substitutions in MHC genes have shown to alter peptide binding capacity, alternative splicing profiles, and for instance, may have impact expression levels. In one NWM species, the common marmoset, expansion of the G/F region was observed, as has been reported earlier (Kono et al. 2014). Our transcription analyses illustrate, however, that subsequent to this expansion most of the paralogous *F* genes were inactivated. This suggests that selection favoured the original function of the *F* gene. In the different primate species, we witnessed varying of mutations that may have affected the composition of the peptide binding groove of *HLA-F* and its orthologues. Most of these genetic alterations are independent effects, that may, however, influence the repertoire of peptides that can be bound.

In comparison to the great apes, the investigated cohorts of macaques were large. In 101 cynomolgus- and 60 rhesus macaques we detected, 31 *Mafa-F* and 22 *Mamu-F* alleles, respectively. Notwithstanding the considerable number of alleles, the level of polymorphism encountered is low, and not exclusively restricted to exon 2 and exon 3. However, the nucleotide differences are more often nonsynonymous in macaques than in humans. For example, the 31 *Mafa-F* alleles, at present archived in the IPD/MHC database, are translated in 22 different proteins, whereas 44 *HLA-F* alleles code for only six proteins. This would suggest that *HLA-F* in the human population is experiencing a more stringent type of selection. The numbers of animals in the other genera of nonhuman primates were too low for meaningful comparisons.

Our results confirm the observation that an R62W substitution, leading to an open-ended groove, is only present in humans and orangutan (Dulberger et al. 2017). Furthermore, in comparison to humans and great apes, the OWM and NWM share a 6-nucleotides-long insertion in exon 2, leading to a protein that is two amino acids longer in the antigen presenting domain. Additional studies on the peptide binding characteristics of the macaque MHC-F allotype may elucidate the effect of this extension on the repertoire of peptides that can be bound.

Earlier genomic studies on the G/F region of the marmoset, in combination with the present findings, show that the *F* gene is duplicated on the marmoset chromosome. However, one gene, *Caja-F4*, may encode a functional product. The other genes/loci are pseudogenes, although most of them are transcribed into mRNA. Our study suggests that another functional *F* gene may be present on the marmoset chromosome, namely, *Caja-F6*. This means that up to four alleles per animal may be present that are translated into proteins. Whereas one or two alleles of the *Caja-F4* gene are expected, the animals Avon and Enwor possess three alleles of this gene. An explanation may be found in the fact that marmosets are born as bone marrow chimeric twins, and blood cells of the twin may still be present in the circulation of an animal.

It may be interesting to date the expansion of the G/F region in the common marmosets. It is known that the *Aotus/Cebus/Saimiri* lineage, to which the grey-bellied night-monkey belongs, radiated from the *Saguinus/Leontopithecus/Callimico/Cebuella/Callithrix* lineage (Opazo et al. 2006). In the latter lineage, the *Saguinus* group, including the cotton-top tamarin, was the first to radiate of. In our panel of tamarins (*Saguinus oedipus*), we found no signs of expansion, and only one *F* gene per haplotype was detected. This indicates that the expansion of the G/F segment took place after the radiation of the *Sanguinus* group, during evolution of the *Leontopithecus/Callimico/Cebuella/Callithrix* lineage. To pinpoint the G/F expansion in NWM evolution, further research on representative animals of the *Leontopithecus/Callimico/Cebulla* lineage is necessary.

This study demonstrates that the ancestry and evolution of a non-classical MHC class I gene such as *HLA-F* can be tracked by way of comparative analyses involving different primate species. Further research is required, however, to determine whether the different orthologous genes in these species execute the same function.

## Electronic supplementary material

Below is the link to the electronic supplementary material.

Supplementary file1 (DOCX 16.9 KB)

Supplementary file2: Suppl. Figure 1: DNA alignment of full-length F-like sequences, detected in this study. The alleles are grouped as great apes, OWM, and NWM. For Chimpanzee (*Patr)* and OWM, only a few representative alleles are included. The Bonobo sequence (*Pan paniscus, Papa*), was downloaded from Genbank. The alleles of sooty mangabey (*Cercocebus atys, Ceat*), an African OWM, were downloaded from the IPD database. The *hyphens* at the start of these sequences mean that this part was not available. The *hyphens* in other alleles refer to deletions in comparison to the human reference sequence. *Lowercase* letters refer to synonymous differences as compared with the HLA-F*01:01, whereas *capitals* indicate nonsynonymous changes. The codon for tryptophan in Human and Orangutan sequences at position 247 is *shaded*. The *bold* T in exon 4 of the sequence Caja-F7*07:01 N represents a double T at this position. Inclusion of the extra T would disrupt the alignment. (PDF 11.6 MB)

Supplementary file3: Suppl. Figure 2: DNA alignment of full-length F-like sequences of cynomolgus (*Mafa*) and rhesus (*Mamu*) macaques. All allotypes found in this study are included. *Lowercase* letters refer to synonymous differences as compared with consensus sequence, whereas *capitals* indicate nonsynonymous changes. (PDF 7.26 MB)
