# Supplementary material for: Evolution of HLA-F and its orthologues in primate species: a complex tale of conservation, diversification and inactivation
Source: Immunogenetics. 2020 Nov 12;72(9):475–87. doi: 10.1007/s00251-020-01187-1 (PMC7725694; doi:10.1007/s00251-020-01187-1)
Supplement: Supplementary file 1 — Supplementary file1 (DOCX 16.9 KB) [file 251_2020_1187_MOESM1_ESM.docx]

Supplementary Table 1. Novel alleles found in this study.

| **Allele** | **Accession** | **Animals** | **Remarks** | **Identical to** |
| --- | --- | --- | --- | --- |
| **Great apes** |  |  |  |  |
| *Patr-F*01:01:02* | LT899681 | regina |  |  |
| *Patr-F*01:01:03* | LT899682 | wouter |  |  |
| *Patr-F*01:02:01* | LT899683 | victoria |  |  |
| *Patr-F*01:02:02* | LT899684 | leonne |  |  |
| *Patr-F*01:03* | LT899685 | louise |  |  |
| *Gogo-F*01:01* | LT899686 | GG05 |  |  |
| *Gogo-F*01:03* | LR655823 | Mapasa |  |  |
| *Poab-F*01:01:02* | LS992574 | PPY, Guchi |  |  |
| *Poab-F*01:01:01* | LS992575 | PPY, Jinjing |  |  |
| *Popy-F*01:01:01* | LS992573 | elmar |  |  |
| **Old World monkeys** |  |  |  |  |
| *Mafa-F*02:02:01* | LT841411 | 1112173 | confirmation | *Mamu-F*02:04:02* |
| *Mafa-F*02:02:02* | LT841410 | 1112197 | confirmation | *Mamu-F*02:04:01* |
| *Mafa-F*02:02:03* | LT841395 | 0301041 |  | DQ367725 |
| *Mafa-F*02:02:04* | LT841396 | 1105195 |  |  |
| *Mafa-F*02:02:05* | LT841397 | 1105180 |  |  |
| *Mafa-F*02:03:01* | LT841398 | 1207004 | confirmation | *Mamu-F*02:07:01* |
| *Mafa-F*02:03:02* | LT841399 | 1208004 |  |  |
| *Mafa-F*02:03:03* | LT899407 | 0612502 |  | *Mamu-F*02:07:03* |
| *Mafa-F*02:04:01* | LT899399 | C0605012 | confirmation |  |
| *Mafa-F*02:04:02* | LT899406 | C1201013 |  | *Mane-F*02:01:01* |
| *Mafa-F*02:06:01* | LT841400 | 1205014 | confirmation |  |
| *Mafa-F*02:06:02* | LT899402 | Y1303004 |  |  |
| *Mafa-F*02:07* | LT841401 | Y9704122 | confirmation |  |
| *Mafa-F*02:08* | LT841402 | 1112383 |  | *Mamu-F*02:08:02* |
| *Mafa-F*02:09:01* | LT841403 | Y1202032 |  | *Mamu-F*02:09* |
| *Mafa-F*02:09:02* | LT841404 | 0302111 |  |  |
| *Mafa-F*02:09:03* | LT841405 | ji6132 |  |  |
| *Mafa-F*02:09:04* | LT899412 | Y0609068 |  |  |
| *Mafa-F*02:10* | LT841406 | Y9402012 |  | *Mamu-F*02:12* |
| *Mafa-F*02:11* | LT841407 | ji401D |  |  |
| *Mafa-F*02:12* | LT841408 | 07050704 |  |  |
| *Mafa-F*02:13* | LT841409 | 0604541 |  |  |
| *Mafa-F*02:14* | LT899400 | 0209228 |  |  |
| *Mafa-F*02:15* | LT899401 | Y0604025 |  |  |
| *Mafa-F*02:16* | LT899403 | Y1202028 |  |  |
| *Mafa-F*02:17* | LT899404 | 1112186 |  |  |
| *Mafa-F*02:18* | LT899405 | 1205019 |  |  |
| *Mafa-F*02:19* | LT899408 | Y1202029 |  |  |
| *Mafa-F*02:20* | LT899409 | Y1303006 |  | *Mamu-F*02:18* |
| *Mafa-F*02:21* | LT899410 | Y0601109 |  |  |
| *Mafa-F*02:22* | LT899411 | Y0611035 | 3' partly |  |
| *Mamu-F*02:01* | LT899414 | 8769 | confirmed, ind |  |
| *Mamu-F*02:02* | LT899415 | r05077 | confirmed, ind |  |
| *Mamu-F*02:03:01* | LT899416 | 96084 | confirmed, ind chi |  |
| *Mamu-F*02:03:02* | LT899417 | 9910010Y | chi |  |
| *Mamu-F*02:04:01* | LT899418 | 9011 | confirmed, ind chi | *Mafa-F*02:02:02* |
| *Mamu-F*02:04:02* | LT899419 | 9309009Y | chi | *Mafa-F*02:02:01* |
| *Mamu-F*02:04:03* | LT899420 | 95512 | chi |  |
| *Mamu-F*02:07:01* | LT899421 | r01054 | ind chi | *Mafa-F*02:03:01* |
| *Mamu-F*02:07:02* | LT899422 | 9202008Y | chi |  |
| *Mamu-F*02:07:03* | LT899423 | 9202008Y | chi | *Mafa-F*02:03:03* |
| *Mamu-F*02:08:01* | LT899424 | r03081 | ind |  |
| *Mamu-F*02:08:02* | LT899425 | 0004012Y | chi | *Mafa-F*02:08* |
| *Mamu-F*02:09* | LT899426 | r09151 | ind | *Mafa-F*02:09:01* |
| *Mamu-F*02:10* | LT899427 | r04060 | ind |  |
| *Mamu-F*02:11* | LT899428 | r01054 | ind |  |
| *Mamu-F*02:12* | LT899429 | 99073 | chi | *Mafa-F*02:10* |
| *Mamu-F*02:13* | LT899430 | 98661 | chi |  |
| *Mamu-F*02:14* | LT899431 | 09203 | chi |  |
| *Mamu-F*02:15* | LT899432 | 0705008y | chi |  |
| *Mamu-F*02:16* | LT899433 | 0808004Y | chi |  |
| *Mamu-F*02:17* | LT899434 | 09057 | chi |  |
| *Mamu-F*02:18* | LT899435 | 98354 | chi | *Mafa-F*02:20* |
| *Mane-F*02:01:01* | LT899670 | 840, CT2C |  | *Mafa-F*02:04:02* |
| *Mane-F*02:01:02* | LT899671 | V676, 1X |  |  |
| *Mane-F*02:01:03* | LT899672 | AV6V, 78W |  |  |
| *Mane-F*02:01:04* | LT899673 | 58Y, 854 |  |  |
| *Mane-F*02:02:01* | LT899674 | AT4K, 15Y |  |  |
| *Mane-F*02:02:02* | LT899675 | AV6V, 69Y |  |  |
| *Mane-F*02:03* | LT899676 | 42R, 1X |  |  |
| *Mane-F*02:04* | LT899677 | 5Y, LJ2 |  |  |
| *Mane-F*02:05* | LT899678 | 67Z, 69Z |  |  |
| *Mane-F*02:06* | LT899679 | 5Y, 854 |  |  |
| *Mane-F*02:07* | LT899680 | 94Z |  |  |
| *Paan-F*02:01:01* | LT899666 | V9929E |  |  |
| *Paan-F*02:01:02* | LT899667 | PA936G |  |  |
| *Paan-F*02:02* | LT899668 | V9813D |  |  |
| *Paan-F*02:03* | LT899669 | K922AD |  |  |
| **New World monkeys** |  |  |  |  |
| *Caja-F1*08:01N* | LR213626 | annabelle, avon |  |  |
| *Caja-F1*08:02N* | LR731936 | Earth, m09122 |  |  |
| *Caja-F4*04:01* | LR213623 | m10018, m11032 |  |  |
| *Caja-F4*04:03* | LT996921 | ergo |  |  |
| *Caja-F4*04:06* | LR723088 | Avon, Enwor |  |  |
| *Caja-F4*04:07* | LR723089 | Avon, Enwor |  |  |
| *Caja-F5*04:02N* | LT996919 | ergo |  |  |
| *Caja-F5*04:04N* | LR213622 | annabelle, m09122 |  |  |
| *Caja-F5*04:05N* | LR213625 | m10115, m11032 |  |  |
| *Caja-F6*06:01* | LT996920 | ergo |  |  |
| *Caja-F6*06:02* | LT996922 | errant |  |  |
| *Caja-F7*07:01N* | LR213624 | ergo, m13024 |  |  |
| *Saoe-F*03:02* | LR656260 | R187, T00-06 |  |  |
| *Saoe-F*03:03* | LR656261 | 95-50, B224 |  |  |
| *Saoe-F*03:04* | LR656262 | 95-07, R231 |  |  |
| *Aole-F*05:01* | LS481192 | A50 |  |  |
| *Aole-F*05:02* | LS481193 | A50 |  |  |
| *Aole-F*05:03* | LS481194 | A0005 |  |  |
| *Aole-F*05:04* | LS481195 | A0005 |  |  |
| *Aole-F*05:05* | LS481196 | A0105 |  |  |

The ENA accession number and a reference animal are given for each allele. Some sequences *confirmed* alleles that were already archived in the IPD database. Alleles that are shared by two macaque species, are listed in the last column.

Ind: allele found in Indian rhesus macaques.

Chi: allele found in Chinese rhesus macaques.
