# Supplementary material for: Evolution of HLA-F and its orthologues in primate species: a complex tale of conservation, diversification and inactivation
Source: Immunogenetics. 2020 Nov 12;72(9):475–87. doi: 10.1007/s00251-020-01187-1 (PMC7725694; doi:10.1007/s00251-020-01187-1)
Supplement: Supplementary file 2 — Supplementary file2: Suppl. Figure 1: DNA alignment of full-length F-like sequences, detected in this study. The alleles are grouped as great apes, OWM, and NWM. For Chimpanzee (Patr) and OWM, only a few representative alleles are included. The Bonobo sequence (Pan paniscus, Papa), was downloaded from Genbank. The alleles of sooty mangabey (Cercocebus atys, Ceat), an African OWM, were downloaded from the IPD database. The hyphens at the start of these sequences mean that this part was not available. The hyphens in other alleles refer to deletions in comparison to the human reference sequence. Lowercase letters refer to synonymous differences as compared with the HLA-F*01:01, whereas capitals indicate nonsynonymous changes. The codon for tryptophan in Human and Orangutan sequences at position 247 is shaded. The bold T in exon 4 of the sequence Caja-F7*07:01 N represents a double T at this position. Inclusion of the extra T would disrupt the alignment. (PDF 11.6 MB) [file 251_2020_1187_MOESM2_ESM.pdf]

```
                                        EXON 1                                             EXON 2

                         10        20        30        40        50        60    |    70        80        90       100    
                 ----:----|----:----|----:----|----:----|----:----|----:----|----|:----|----:----|----:----|----:----|----
HLA-F*01:01      ATGGCGCCCCGAAGCCTCCTCCTGCTGCTCTCAGGGGCCCTGGCCCTGACCGATACTTGGGCGG|GCTCCCACTCCTTGAGGTATTTCAGCACCGCTGTGTCGCG
HLA-F*01:02      .....................................T..........................|........................................
Patr-F*01:01:02  ...........................T.........................G........a.|........................................
Patr-F*01:02:01  .....................................................G........a.|........................................
Patr-F*01:03     ...............................T.....................G........a.|............................G...........
Papa-F AF084027  .....................................................G........a.|........................................
Gogo-F*01:01     ...........................T.......................A.G........a.|........................................
Gogo-F*01:03     ...........................T.......................A.G........a.|........................................
Poab-F*01:01:01  .............C.......................................G..c.......|........................................
Popy-F*01:01:02  .............C.......................................G..c.......|........................................
Mamu-F*02:01     .............C..........G............................G..c.......|..............c.......t.................
Mamu-F*02:02     .............C..........G............................G..c.......|..............c.......t.................
Mafa-F*02:02:01  .............C..........G............................G..c.......|..............c.......t.................
Mafa-F*02:03:01  .............C..........G............................G..c.......|..............c.......t.................
Mane-F*02:01:01  .....a.......C..........G............................G..c.......|..............c.......t.................
Mane-F*02:02:01  .....a.......C..........G............................G..c.......|..............c.......t.................
Paan-F*02:01:01  .....a.......C..........G............................G..c.......|..............c.......t.................
Paan-F*02:02     .....a.......C..........G............................G..c.......|..............c.......t................A
Ceat-F*02:01     ------------------------G............................G..c.......|..............c.......t.................
Ceat-F*02:02     ------------------------G............................G..c.......|..............c.......t.................
Saoe-F*03:02     .............C.................TG.........A..........G..c.......|.........................t..t..cA....c..
Saoe-F*03:03     .............C.................TG.........A..........G..c.......|.........................t..t..cA....c..
Saoe-F*03:04     .............C.................TG.........A..........G..c.......|.........................t..t..cA....c..
Aole-F*05:01     .....a..aG...C..................g...a................G..c.......|.t.............A......G........c.....c..
Aole-F*05:02     .....a..aG...C..................g...a................G..c.......|.t.............A......G........c.....c..
Aole-F*05:03     .....a..aG...C..................g...a................G..c.......|.t.............A......G........c.....c..
Aole-F*05:04     .....a..aG...C..................g...a................G..c.......|.t.............A......G........c.....c..
Aole-F*05:05     .....a..aG...C..................g...a................G..c.......|.t.............A......G........c.....c..
Caja-F4*04:01    .....a..a....C...............t..t...............G....G..cCA.....|......................G........c.....c..
Caja-F4*04:03    .....a..a....CT..................t....................G..cCA.....|..............c.......G........c.....c..
Caja-F4*04:06    .....a..a....C...............t..t...............G....G..cCA.....|..............c.......G........c.....c..
Caja-F4*04:07    .....a..a....C...............t..t...............G....G..cCA.....|......................G........c.....c..
Caja-F6*06:01    .....a..a....C...............t..t....................G..cC.T....|......................G........c.....c..
Caja-F6*06:02    .....a..a....C...............t..t....................G..cC.T....|......................G........c.....c..
Caja-F5*04:02N   .....a..a....C...............t..t...............G....G..cCA.....|..............c.......G........c.....c..
Caja-F5*04:04N   .....a..a....C..................t....................G..cCA.....|..............c.......G........c.....c..
Caja-F5*04:05N   .....a..a.T..C...............t..t...............G....G..cCA.....|..............c.......G........c.....c..
Caja-F7*07:01N   .....a..a....T..................t....................G..cCA.....|..............c.......G........c.....c..
Caja-F1*08:01N   ....A........CG...T.......a....TG.........A..........G..c....T..|.............................A.c.....c.C
Caja-F1*08:02N   ....A........CG...T.......a....TG.........A..........G..c....T..|.............................A.c.....c.C
```

EXON 2

```
                       110       120             130       140       150       160       170       180       190       200
                    :----|----:----|---......-:----|----:----|----:----|----:----|----:----|----:----|----:----|----:----|--
HLA-F*01:01         GCCCGGCCGCGGGGAGCCC------CGCTACATCGCCGTGGAGTACGTAGACGACACGCAATTCCTGCGGTTCGACAGCGACGCCGCGATTCCGAGGATGGAGC
HLA-F*01:02         ...................------...............................................................................
Patr-F*01:01:02     ...................------..g................................g...........................................
Patr-F*01:02:01     ...................------..g................................g...........................................
Patr-F*01:03        ...................------..g................................g...........................................
Papa-F AF084027     ...................------..g................................g...........................................
Gogo-F*01:01        ...................------..g................................g...........................................
Gogo-F*01:03        ...................------..g.............................a..g...........................................
Poab-F*01:01:01     ...................------..g....................g...........g...........t...............................
Popy-F*01:01:02     ...................------..g....................g...........g...........t...............................
Mamu-F*02:01        ..........A.......tCGGTACa.g........g...........g...........g...........................................
Mamu-F*02:02        ..........A.......tCAGTACa.g........g...........g...........g...........................................
Mafa-F*02:02:01     ..........A.......tCAGTACa.g........g...........g...........g...........................................
Mafa-F*02:03:01     ..........A.......tCAGTACa.g........g...........g...........g...........................................
Mane-F*02:01:01     ..........A.......tCGGTACa.g........g...........g...........g...........................................
Mane-F*02:02:01     ..........A.......tCGGTACa.g........g...........g...........g...........................................
Paan-F*02:01:01     ..........A.......tCGGTACa.g........g...........g...........g...........................................
Paan-F*02:02        ...g......A.......tCGGTACa.g........g...........g...........g...........................................
Ceat-F*02:01        ..........A.......tCGGTACa.g........g...........g...........g...........................................
Ceat-F*02:02        ..........A.......tCGGTACa.g........g...........g...........g...........................................
Saoe-F*03:02        ...t...............CGATACa.g.......GA....GC.....g..t.......TG...G..a...............A.C...G..............
Saoe-F*03:03        ...t...............CGATACa.g.......GA....GC.....g..t.......TG...G..a...............A.C...G..............
Saoe-F*03:04        ...t...............CGATACa.g.......GA....GC.....g..t.......TG...G..a...............A.C...G..............
Aole-F*05:01        ...t......C........CGATACa.g........a....GC.....g..........TG...A.......t................G..............
Aole-F*05:02        ...t......C........CGATACa.g........a....GC.....g..........TG...A.......t................G..............
Aole-F*05:03        ...t......CA.......CGATACa.g........a....GC.....g..........TG...A.......t................G..............
Aole-F*05:04        ...t......C........CGATACa.g........a....GC.....g..........TG...A.......t................G..............
Aole-F*05:05        ...t......C........CGATACa.g........a....GC.....g..........TG...A.......t................G..............
Caja-F4*04:01       ...t............T..AGATACa.g........a....GC.....g...........g...A.......t............CA..G.....T........
Caja-F4*04:03       ...t............T..AGATACa.g........a....GC.....g...........g...A.......t............CA..G.....T........
Caja-F4*04:06       ...t............T..AGATACa.g........a....GC.....g...........g...A.......t............CA..G.....T........
Caja-F4*04:07       ...t............T..AGATACa.g........a....GC.....g...........g...A.......t............CA..G.....T........
Caja-F6*06:01       ...t............T..AGATACa.g........a....GC.....g...........g...A.......t............CA..G.....T........
Caja-F6*06:02       ...t............T..AGATACa.g........a....GC.....g...........g...A.......t............CA..G.....T........
Caja-F5*04:02N      ...t............T..AGATACa.g........a....GC.....g...........g...........t............CA..G.....T........
Caja-F5*04:04N      ...t............T..AGATACa.g........a....GT.....g...............A.......t............CA..G.....T........
Caja-F5*04:05N      ...t............T..AGATACa.g........a....GC.....g...........g...........t............CA..G.....T........
Caja-F7*07:01N      ...t............T..AGATACa.g........a....GC.....g...T...................t............CA..G.....T........
Caja-F1*08:01N      ..........T........CGATACa.g.......GA....GC.....g...........gA..G........................G..............
Caja-F1*08:02N      ..........T........CGATACa.g.......GA....GC.....g...........gA..G........................G..............
```

EXON 2

```
                          210       220       230       240       250       260       270       280       290       300
                     --:----|----:----|----:----|----:----|----:----|----:----|----:----|----:----|----:----|----:----|----:-
HLA-F*01:01          CGCGGGAGCCGTGGGTGGAGCAAGAGGGGCCGCAGTATTGGGAGTGGACCACAGGGTACGCCAAGGCCAACGCACAGACTGACCGAGTGGCCCTGAGGAACCTG
HLA-F*01:02          ........................................................................................................
Patr-F*01:01:02      .t..........................................A................g..........................................
Patr-F*01:02:01      .t..........................................A..........a.....g..........................................
Patr-F*01:03         .t.........................................aA................g..........................................
Papa-F AF084027      .t..........................................A..........a.....g..........................................
Gogo-F*01:01         .t...................................c......A...........................................................
Gogo-F*01:03         .t..........................................A..............................................a............
Poab-F*01:01:01      ......C.........................................................................................A.......
Popy-F*01:01:02      ......C.........................................................................................A.......
Mamu-F*02:01         ......C.....................................C..............................G........................G...
Mamu-F*02:02         ....a.C.....................................C..............................G........................G...
Mafa-F*02:02:01      ....a.C.....................................C..............................G........................G...
Mafa-F*02:03:01      ....a.C.....................................C..............................G........................G...
Mane-F*02:01:01      ......C.....................................C..............................G........................G...
Mane-F*02:02:01      ......C.....................................C..............................G........................G...
Paan-F*02:01:01      ....a.C.....................................C..............................G........................G...
Paan-F*02:02         ....a.C.......................a............C..............................G........................G..a
Ceat-F*02:01         ....a.C..A.....................a............C..............................G........................G...
Ceat-F*02:02         ....a.C.......................a............C..............................G........................G...
Saoe-F*03:02         ......C...............g...A.....G...........GACCAG...C..GT..T............................AAA...c....A...
Saoe-F*03:03         ......C...............g...A.....G...........GACCAG...C..GT..T............................AAA...c....A...
Saoe-F*03:04         ......C...............g...A.....G...........GACCAG...C..GT..T............................AAAA..c....A...
Aole-F*05:01         ......C...............g...A.....G..........CC..---------G.G.T...............a............AA....c...T....
Aole-F*05:02         ......C...............g...A.....G..........CC..---------G.G.T...............a............AA....c...T....
Aole-F*05:03         ......C...............g...A.....G..........CC..---------G.G.T...............a............AA....c...T....
Aole-F*05:04         ......C...............g...A.....G..........CC..---------G.G.T...............a............AA....c...T....
Aole-F*05:05         ......C...............g...A.....G..........CC..---------G.G.T...............a............AA....c...T....
Caja-F4*04:01        ......C...............g...A.A...G..........CC..---------G.G.T.....AG.G......a......a....aAA....c...T....
Caja-F4*04:03        ......CA..............g...A.....G..........CC..---------G.G.T.....AG.G......a...........aAA....c...T....
Caja-F4*04:06        ......C...............g...A.A...G..........CC..---------G.G.T.....AG.G......a...........aAA....c...T....
Caja-F4*04:07        ......C...............g...A.....G..........CC..---------G.G.T.....AG.G......a...........aAA....c...T....
Caja-F6*06:01        ......C...............g...A.....G..........CC..---------G.G.T.....AG.G......a...........aAA....c...T....
Caja-F6*06:02        ......C....A..........g...A.....G..........CC..---------G.G.T.....AG.G......a...........aAA....c...T....
Caja-F5*04:02N       ......C...............g...A.....G...........C..---------G.G.T...a.AG.T......a............AA....c...T....
Caja-F5*04:04N       ......CA..............g...A.....G..........CC..---------G.G.T.....AG.G......a...........aAA....c...T....
Caja-F5*04:05N       ......C...............g...A.....G...........C..---------G.G.T...a.AG.T......a............AA....c...T....
Caja-F7*07:01N       ......C...............g...A.....G...........C..---------G.G.T...a.AG.G......a............AA....c...T....
Caja-F1*08:01N       ......CA..............g.....................GA.GAG...CT.GTA.T........G......a......a.....AAA...c...T.GCT
Caja-F1*08:02N       ......CA..............g.........G...........GA.GAG...CT.GTA.T........G......a......a.....AAA...c...T.GCT
```

EXON 3

```
                       310            320            330    |   340            350            360            370            380            390            400            410
                    ---|----:----|----:----|----|:----|----:----|----:----|----:----|----:----|----:----|----:----|----:----|
HLA-F*01:01         CTCCGCCGCTACAACCAGAGCGAGGCTG|GGTCTCACACCCTCCAGGGAATGAATGGCTGCGACATGGGGCCCGACGGACGCCTCCTCCGCGGGTATCACCAGCA
HLA-F*01:02         ............................|............................................................................
Patr-F*01:01:02     ..........................c.|..........................................................t.................
Patr-F*01:02:01     ..........................c.|............................................................................
Patr-F*01:03        ..........................c.|............................................................................
Papa-F AF084027     ..........................c.|............................................................................
Gogo-F*01:01        ..........................c.|..........................................................t.................
Gogo-F*01:03        ..........................c.|..........................................................t.................
Poab-F*01:01:01     ..........................c.|.a..........................................................................
Popy-F*01:01:02     ..........................c.|.a..........................................................................
Mamu-F*02:01        ....T.....................a.|.........................c..................................................
Mamu-F*02:02        ....T.....................c.|.........................c..................................................
Mafa-F*02:02:01     ....T.....................c.|............................................................................
Mafa-F*02:03:01     ....T.....................c.|.........................c..........................t.......................
Mane-F*02:01:01     ....T.....................c.|.........................c..................................................
Mane-F*02:02:01     ....T.....................c.|.........................c..................................................
Paan-F*02:01:01     ....T...a.................c.|............................................................................
Paan-F*02:02        ....T...a.................c.|............................................................................
Ceat-F*02:01        ....T...a.................c.|............................................................................
Ceat-F*02:02        ....T...a.................c.|............................................................................
Saoe-F*03:02        ......TA......t..........GC.|.....................C............tG....a.....t......T..........a...........
Saoe-F*03:03        ......TA......t..........GC.|.....................C............tG....a.....t......T..........a...........
Saoe-F*03:04        ......TA......t..........GC.|.....................C............tG....a.....t......T..........a...........
Aole-F*05:01        ......TA....................|..................................tG....a..t..t......T..........a...........
Aole-F*05:02        ......TA....................|..................................tG....a..t..t......T..........a...........
Aole-F*05:03        ......TA....................|..................................tG....a..t..t......T..........a...........
Aole-F*05:04        ......TA....................|..................................tG....a..t..t......T..........a...........
Aole-F*05:05        ......TA....................|..................................tG....a..t..t......T..........a...........
Caja-F4*04:01       ......TA................A.C.|..........g.......................tG....a..g..t......T....t..a..............
Caja-F4*04:03       ......TA................A.C.|.........AG.......................tG....a..g..t......T....t..a..............
Caja-F4*04:06       ......TA................A.C.|.........AG.......................tG....a..g..t......T....t..a..............
Caja-F4*04:07       ......TA................A.C.|..........g.......................tG....a..g..t......T....t..a..............
Caja-F6*06:01       ......TA................A.C.|..........g.......................tG....a.....t......T.T..t..a..............
Caja-F6*06:02       ......TA................A.C.|..........g.......................tG....a.....t......T.T..t..a..............
Caja-F5*04:02N      ......TA................A.C.|........--.......T................tG....a..g..t......T....t..a..............
Caja-F5*04:04N      ......TA................A.C.|---------------------------------------------------------------.............
Caja-F5*04:05N      ......TA................A.C.|........--.......T................tG....a..g..t......T....t..a..............
Caja-F7*07:01N      ......TA................ATC.|.........AG.......................tG.A..a.....t......T....t..a..............
Caja-F1*08:01N      ...G..TA..................c.|........--.....................A..tG....a.....t....A.T......................
Caja-F1*08:02N      ...G..TA..................c.|........--.....................A..tG....a.....t....A.T......................
```

EXON 3

```
                          420       430       440       450       460       470       480       490       500       510
                   ----:----|----:----|----:----|----:----|----:----|----:----|----:----|----:----|----:----|----:----|----
HLA-F*01:01        CGCGTACGACGGCAAGGATTACATCTCCCTGAACGAGGACCTGCGCTCCTGGACCGCGGCGGACACCGTGGCTCAGATCACCCAGCGCTTCTATGAGGCAGAGG
HLA-F*01:02        ........................................................................................................
Patr-F*01:01:02    ........................................................................................................
Patr-F*01:02:01    ........................................................................................................
Patr-F*01:03       ........................................................................................................
Papa-F AF084027    ........................................................................................................
Gogo-F*01:01       ........................................................................................................
Gogo-F*01:03       ........................................................................................................
Poab-F*01:01:01    ........................................................................................................
Popy-F*01:01:02    ............................................................c...........................................
Mamu-F*02:01       ...c.....................................................c........g..a....G.............................
Mamu-F*02:02       ...c.....................................................c........g..a....G.............................
Mafa-F*02:02:01    ...c.....................................................c........g..a....G.............................
Mafa-F*02:03:01    ...c.....................................................c........g..a....G.............................
Mane-F*02:01:01    ...c.....................................................c........g..a....G.............................
Mane-F*02:02:01    ...c.....................................................c........g..a....G.............................
Paan-F*02:01:01    ...c.....................................................c........g..a....G...........................T.
Paan-F*02:02       ...c.....................................................c........g..a....G...........................T.
Ceat-F*02:01       ...c..................................................t..c........g..a....G...........................T.
Ceat-F*02:02       ...c..................................................t..c........g..a....G...........................T.
Saoe-F*03:02       ...c..............c.........................................a.....a.C...................A.G......T.....A
Saoe-F*03:03       ...c..............c.........................................a.....a.C...................A.G......T.....A
Saoe-F*03:04       ...c......T.......c.........................................a.....a.C...................A.G......T.....A
Aole-F*05:01       ...c............................................................GTG.C.............A.....AAG.GG.........A
Aole-F*05:02       ...c............................................................GTG.C.............A.....AAG.GG.........A
Aole-F*05:03       ...c............................................................GTG.C.............A.....AAG.GG.........A
Aole-F*05:04       ...c............................................................GTG.C.............A.....AAG.GG.........A
Aole-F*05:05       ...c............................................................GTG.C.............A.....AAG.GG.........A
Caja-F4*04:01      ...c..............c...............................................a.CA..................AAG.GG.........A
Caja-F4*04:03      ...c..t...........c...............................................a.CA..................AAG.GG.........A
Caja-F4*04:06      ...c..t...........c...............................................a.CA..................AAG.GG.........A
Caja-F4*04:07      ...c..............c...............................................a.CA..................AAG.GG.........A
Caja-F6*06:01      ...c..............c..t............................................a.CA..................AAG.GG.........A
Caja-F6*06:02      ...c..............c..t............................................a.CA..................AAG.GG.........A
Caja-F5*04:02N     ...c..............c...............................................a.CA..................AAG.GG.........A
Caja-F5*04:04N     ...c..t...........c..........................t....................a.CA..........A.......AAG.GG.........A
Caja-F5*04:05N     ...c..............c...............................................a.CA..................AAG.GG.........A
Caja-F7*07:01N     ...c..............c...............................................a.CA..................AAG.GG.........A
Caja-F1*08:01N     ...c..............c...............A....................A.......AGTG.C............g......AAG.GG.........A
Caja-F1*08:02N     ...c..............c...............A....................A.......AGTG.C............g......AAG.GG.........A
```

EXON 3

```
                        520       530       540       550       560       570       580       590       600       610|
                     :----|----:----|----:----|----:----|----:----|----:----|----:----|----:----|----:----|----:----||----:---
HLA-F*01:01          AATATGCAGAGGAGTTCAGGACCTACCTGGAGGGCGAGTGCCTGGAGTTGCTCCGCAGATACTTGGAGAATGGGAAGGAGACGCTACAGCGCGCAG|ATCCTCCA
HLA-F*01:02          ................................................................................................|........
Patr-F*01:01:02      ......................................................................c.........................|........
Patr-F*01:02:01      ......................................................................c.........................|........
Patr-F*01:03         ......................................................................c.........................|........
Papa-F AF084027      ......................................................................c.........................|........
Gogo-F*01:01         ......................................................................c.........................|........
Gogo-F*01:03         ......................................................................c.........................|........
Poab-F*01:01:01      .....................T.....................a..................c.......c.........................|....c...
Popy-F*01:01:02      .....................T.....................a..................c.......c.........................|....c...
----------------------------------------------------------------------------------------------------------------------------------
Mamu-F*02:01         .....................T........................................c.......c.........................|........
Mamu-F*02:02         ..............................................................c.......c.........................|........
Mafa-F*02:02:01      ..............................................................c.......c.........................|........
Mafa-F*02:03:01      ..............................................................c.......c.........................|........
Mane-F*02:01:01      ..............................................................c.......c.........................|........
Mane-F*02:02:01      ..............................................................c.......c.........................|........
Paan-F*02:01:01      ..............................................................c.......c.........................|........
Paan-F*02:02         ..............................................................c.......c.........................|........
Ceat-F*02:01         ..............................................................c.......c.........................|........
Ceat-F*02:02         ..............................................................c.......c.........................|........
----------------------------------------------------------------------------------------------------------------------------------
Saoe-F*03:02         ...................C.........A......G....G......G.....A.......c.......c..............g........t.|T.......
Saoe-F*03:03         ...................C.........A......G....G......G.....A.......c.......c..............g........t.|T.......
Saoe-F*03:04         ...................C.........A......G....G......G.....A.......c.......c..............g........t.|T.......
Aole-F*05:01         ...................C.........A......G....G......G.....A......tc.......c..............g........g.|........
Aole-F*05:02         ...................C.........A......G....G......G.....A......tc.......c..............g........g.|........
Aole-F*05:03         ...................C.........A......G....G......G.....A......tc.......c..............g........g.|........
Aole-F*05:04         ...................C.........A......G....G......G.....A......tc.......c..............g........g.|........
Aole-F*05:05         ...................C.........A......G....G......G.....A......tc.......c..............g........g.|...T....
Caja-F4*04:01        ...................C.........A......CA...G......G.....AT......c.......c..............g......A.G.|........
Caja-F4*04:03        ...................C.........A.....AGA...G......G.....AT......c.......c..............g......A.G.|........
Caja-F4*04:06        ...................C.........A.....AGA...G......G.....AT......c.......c..............g......A.G.|........
Caja-F4*04:07        ...................C.........A......CA...G......G.....AT......c.......c..............g......A.G.|........
Caja-F6*06:01        ...................C.........A......GA...A......G.....AT......c.......c............t.g.CA...A.G.|........
Caja-F6*06:02        ...................C.........A......GA...A......G.....AT......c.......c............t.g.CA...A.G.|........
Caja-F5*04:02N       ...................C.........A.....AGA...G......G.....A.......c.......c..............g......A.G.|........
Caja-F5*04:04N       ...................C.........A.....AGA...G......G.....A.......c.......c..............g......A.G.|........
Caja-F5*04:05N       ...................C.........A.....AGA...G......G.....A.......c.......c..............g......A.G.|........
Caja-F7*07:01N       ...................T.........A......G....G......G.....A.......c.......c..............g..a...A.G.|........
Caja-F1*08:01N       .............a.....C.....................G......G.....A..---------------------------------------|........
Caja-F1*08:02N       .............a.....C.....................G......G.....A..---------------------------------------|........
```

EXON 4

```
                    620       630       640       650       660       670       680       690       700       710       720
                    -|----:----|----:----|----:----|----:----|----:----|----:----|----:----|----:----|----:----|----:----|--
HLA-F*01:01         AAGGCACACGTTGCCCACCACCCCATCTCTGACCATGAGGCCACCCTGAGGTGCTGGGCCCTGGGCTTCTACCCTGCGGAGATCACGCTGACCTGGCAGCGGGA
HLA-F*01:02         ........................................................................................................
Patr-F*01:01:02     .........A..............................................................................................
Patr-F*01:02:01     ........................................................................................................
Patr-F*01:03        ........................................................................................................
Papa-F AF084027     ........................................................................................................
Gogo-F*01:01        ........................................................................................................
Gogo-F*01:03        ........................................................................................................
Poab-F*01:01:01     .........A...................................................................c...........c..............
Popy-F*01:01:02     .........A...................................................................c...........c..............
--------------------------------------------------------------------------------------------------------------------------------
Mamu-F*02:01        ..................................G..........................................c........a.................
Mamu-F*02:02        ..................................G..........................................c........a.................
Mafa-F*02:02:01     ..................................G..........................................c........a.................
Mafa-F*02:03:01     ..................................G..........................................c........a.................
Mane-F*02:01:01     ......................A...........G..........................................c........a.................
Mane-F*02:02:01     ......................A...........G..........................................c........a.................
Paan-F*02:01:01     ..................................G..........................................c........a.................
Paan-F*02:02        ..................................G..........................................c........a.................
Ceat-F*02:01        ..................................G..........................................c........a.................
Ceat-F*02:02        ..................................G..........................................c........a.................
--------------------------------------------------------------------------------------------------------------------------------
Saoe-F*03:02        ...........................................T.......................................t.T.............a....
Saoe-F*03:03        ...........................................T.......................................t.T.............a....
Saoe-F*03:04        ...........................................T.......................................t.T.............a....
Aole-F*05:01        ..a.........................................................................G.........a.................
Aole-F*05:02        ..a...................................................................................a.................
Aole-F*05:03        ..a..................T......................................................G.........a.................
Aole-F*05:04        ..a..................T......................................................G.........a.................
Aole-F*05:05        ..a.........................................................................G.........a.................
Caja-F4*04:01       .................t..........................................t...............T........T.............a....
Caja-F4*04:03       .................t..........................a...............t...............T........T.............a....
Caja-F4*04:06       .................t..........................a...............t...............T........T.............a....
Caja-F4*04:07       .................t..........................................t...............T........T.............a....
Caja-F6*06:01       .................t................................a.........t...............T........T.............a....
Caja-F6*06:02       .................t................................a.........t...............T........T.............a....
Caja-F5*04:02N      .................t..........................................t...............T........T.............a....
Caja-F5*04:04N      .................t..........................................t...............T........T.............a....
Caja-F5*04:05N      .................t..........................................t...............T........T.............a....
Caja-F7*07:01N      .................t............................T...a.........t...............T........T.............a....
Caja-F1*08:01N      .................t..........................................t...............T........T.............a....
Caja-F1*08:02N      .................t..........................................t...............T........T.............a....
```

EXON 4

```
                          730       740       750       760       770       780       790       800       810       820
                     --:----|----:----|----:----|----:----|----:----|----:----|----:----|----:----|----:----|----:----|----:-
HLA-F*01:01          TGGGGAGGAACAGACCCAGGACACAGAGCTTGTGGAGACCAGGCCTGCAGGGGATGGAACCTTCCAGAAGTGGGCCGCTGTGGTGGTGCCTTCTGGAGAGGAAC
HLA-F*01:02          ........................................................................................................
Patr-F*01:01:02      ...........................................................A...............t............................
Patr-F*01:02:01      c.......................................................................................................
Patr-F*01:03         c..........................................................................t............................
Papa-F AF084027      c..........................................................A............................................
Gogo-F*01:01         ........................................................................................................
Gogo-F*01:03         ........................................................................................................
Poab-F*01:01:01      ..............................................A.........................................................
Popy-F*01:01:02      ..............................................A.........................................................
Mamu-F*02:01         c.......................g..................................................a.................c........g.
Mamu-F*02:02         c.......................g..................................................a.................c........g.
Mafa-F*02:02:01      c.......................g..................................................a.................c........g.
Mafa-F*02:03:01      c.......................g..................................................a.................c........g.
Mane-F*02:01:01      c.......................g..................................................a.................c........g.
Mane-F*02:02:01      c.......................g..................................................a.................c........g.
Paan-F*02:01:01      c.......................g..................................................a.................c........g.
Paan-F*02:02         c.......................g..................................................a.................c........g.
Ceat-F*02:01         c.......................g..................................................a.................c........g.
Ceat-F*02:02         ........................g..................................................a.................c........g.
Saoe-F*03:02         ...a.....T.................a...............................A...............a.............T......g.....g.
Saoe-F*03:03         ...a.....T.................a...............................................a.............T......g.....g.
Saoe-F*03:04         ...a.....T.................a...............................A...............a.............T......g.....g.
Aole-F*05:01         .........C.............TG..a...............................................g.............T......g.....g.
Aole-F*05:02         .........C.............TG..a...............................................a.............T......g.....g.
Aole-F*05:03         .........C.............TG..a...............................................g.............T......g.....g.
Aole-F*05:04         .........C.............TG..a...............................................g.............T......g.....g.
Aole-F*05:05         .........C.............TG..a...............................................g.............T......g.....g.
Caja-F4*04:01        ...a.....T.................................................................a.............T......g.....gG
Caja-F4*04:03        ...a.....T.................................................................a.............T......g.....gG
Caja-F4*04:06        ...a.....T.................................................................a.............T......g.....gG
Caja-F4*04:07        ...a.....T.................................................................a.............T......g.....gG
Caja-F6*06:01        ...a.....T.................................................................a.............T......g.....g.
Caja-F6*06:02        ...a.....T.................................................................a.............T......g.....g.
Caja-F5*04:02N       ...a.....T.................................................................a.....a.......T......g.....gG
Caja-F5*04:04N       ...a.....T.................................................................a.............T......g.....gG
Caja-F5*04:05N       ...a.....T.................................................................a.....a.......T......g.....gG
Caja-F7*07:01N       ...a.....T.................................................................a.............T..T...g.....g.
Caja-F1*08:01N       ...a.....T.................................................A...............a.............T......g.....g.
Caja-F1*08:02N       ...a.....T.................................................A...............a.............T......g.....g.
```

```
                                        EXON 4                                                     EXON 5

                   830       840       850       860       870       880    |  890       900       910       920       930
                ---|----:----|----:----|----:----|----:----|----:----|----:-|---|----:----|----:----|----:----|----:----|
HLA-F*01:01     AGAGATACACATGCCATGTGCAGCACGAGGGGCTGCCCCAGCCCCTCATCCTGAGATGGG|AGCAGTCTCCCCAGCCCACCATCCCCATCGTGGGCATCGTTGCT
HLA-F*01:02     ............................................................|............................................
Patr-F*01:01:02 ..........g.....................................C...........|............................................
Patr-F*01:02:01 ..........g.....................................C...........|............................................
Patr-F*01:03    .........TG.....................................C...........|............................................
Papa-F AF084027 ..........g.....................................C...........|............................................
Gogo-F*01:01    ..........g.....................................C...........|............................................
Gogo-F*01:03    ..........g.....................................C...........|............................................
Poab-F*01:01:01 ..........c...............A...........G.........C...........|............................................
Popy-F*01:01:02 ..........c...............A...........G.........C...........|............................................
-------------------------------------------------------------------------------------------------------------------------
Mamu-F*02:01    ..........g...........a.....a...................C...........|..TC....T.T.................t...............
Mamu-F*02:02    ..........g...........a.....a.......T...........C...........|..TC....T.T.................t...............
Mafa-F*02:02:01 ..........g...........a.....a...................C...........|..TC....T.T.................t...............
Mafa-F*02:03:01 ..........g...........a.....a...................C...........|..TC....T.T.................t...............
Mane-F*02:01:01 ..........g...........a.....a...................C...........|..TC....T.T.................t...............
Mane-F*02:02:01 ..........g...........a.....a...................C...........|..TC....T.T.................t...............
Paan-F*02:01:01 ..........c...T.......a.....a...................C...........|...C....T.T...........t.....t...............
Paan-F*02:02    ..........c...T.......a.....a...................C...........|...C....T.T...........t.....t...............
Ceat-F*02:01    ..........c...T.......a.....a...................C...........|...C....T.T...........t.....t...............
Ceat-F*02:02    ..........c...T.......a.....a...................C...........|...C....T.T...........t.....t...............
-------------------------------------------------------------------------------------------------------------------------
Saoe-F*03:02    ..c.......c..........................aG..T......C...........|...CA...T...........G...T....A........AC....
Saoe-F*03:03    ..c.......c..........................aG..T......C...........|...CA...T...........G...T....A........AC....
Saoe-F*03:04    ..c.......c..........................aG..T......C...........|...CA...T...........G...T....A........AC....
Aole-F*05:01    ..c.g.....c..........................aG..A......C...........|...CA...T..........tG................GA.....
Aole-F*05:02    ..c.g.....c..........................aG..A......C...........|...CA...T..........tG................GA.....
Aole-F*05:03    ..c.g.....c..........................aG..A......C...........|...CA...T..........tG................GA.....
Aole-F*05:04    ..c.g.....c..........................aG..A......C...........|...CA...T..........tG................GA.....
Aole-F*05:05    ..c.g.....c..........................aG..A......C...........|...CA...T..........tG................GA.....
Caja-F4*04:01   ..c.......c..........................aG.........C...........|...CA...T...............T...................
Caja-F4*04:03   ..c.......c..........................aG.........C...........|...CA...T...............T...................
Caja-F4*04:06   ..c.......c..........................aG.........C...........|...CA...T...............T...................
Caja-F4*04:07   ..c.......c..........................aG.........C...........|...CA...T...............T...................
Caja-F6*06:01   ..c.......c..........G...............aG.......t.C.....A.....|...CA...T...........G...T...................
Caja-F6*06:02   ..c.......c..........G...............aG.......t.C.....A.....|...CA...T...........G...T...................
Caja-F5*04:02N  ..c.......c.........A................aG.........C.......C...|...CA...T...............T...................
Caja-F5*04:04N  ..c.......c..........................aG.........C...........|...CA...T...............T...................
Caja-F5*04:05N  ..c.......c.........A................aG.........C.......C...|...CA...T...............T...................
Caja-F7*07:01N  ..c.......c.........A................aG.........C.....A.....|...CA...T...............T...................
Caja-F1*08:01N  ..c.......c..........................aG.........C...........|...CA...T...........G......A.A........A.....
Caja-F1*08:02N  ..c.......c..........................aG.........C...........|...CA...T...........G......A.A........A.....
```

```
                                      EXON 5                                                 EXON 6

                           940       950       960       970       980       990       1000          |    1010      1020
                   ----:----|----:----|----:----|----:----|----:----|----:----|----:----|---.........|-:----|----:----|----:
HLA-F*01:01        GGCCTTGTTGTCCTTGGAGCTGTGGTCACTGGAGCTGTGGTCGCTGCTGTGATGTGGAGGAAGAAGAGCTCAG---------|ATAGAAACAGAGGGAGCTACTC
HLA-F*01:02        .........................................................................---------|......................
Patr-F*01:01:02    .........................................................................---------|......................
Patr-F*01:02:01    .................................................................C.......---------|......................
Patr-F*01:03       .........................................................................---------|......................
Papa-F AF084027    .................................................................C.......---------|......................
Gogo-F*01:01       ...................................................................T.....---------|......................
Gogo-F*01:03       .........................................................................---------|......................
Poab-F*01:01:01    ..................................................a......................---------|......................
Popy-F*01:01:02    ..................................................a......................---------|......................
Mamu-F*02:01       .....g.C......a.C..T......t.......................a..........GA..........---------|......................
Mamu-F*02:02       .....g.C......a.C..T......t.......................a..........GA..........---------|......................
Mafa-F*02:02:01    .....g.C......a.C..T......t.......................a..........GA..........---------|......................
Mafa-F*02:03:01    .....g.C......a.C..T......t...............A.......a..........GA..........---------|......................
Mane-F*02:01:01    .....g.C......a.C..T..............................a.......A..G...........---------|......................
Mane-F*02:02:01    .....g.C......a.C..T..............................a..........GA..........---------|......................
Paan-F*02:01:01    .....g.C......a.C..T................A.............a..........G...........---------|......................
Paan-F*02:02       .....g.C......a.C..T................A.............a..........G...........---------|......................
Ceat-F*02:01       .....g.C......a.C..T................A....t........a..........G...........---------|......................
Ceat-F*02:02       .....g.C......a.C..T................A....t........a..........G...........---------|......................
Saoe-F*03:02       ..tG.G...........................------------......................T..GGTTGGGAAGGG|......................
Saoe-F*03:03       ..tG.G...........................------------......................T..GGTTGGGAAGGG|......................
Saoe-F*03:04       ..tG.G...........................------------......................T..GGTTGGGAAGGG|......................
Aole-F*05:01       .....g............T...........---------------.G................---.T.....---------|............A.........
Aole-F*05:02       .....g............T...........---------------.G................---.T.....---------|............A.........
Aole-F*05:03       .....g............T...........---------------.G................---GT.....---------|............A.........
Aole-F*05:04       .....g............T...........---------------.G................---.T.....---------|............A.........
Aole-F*05:05       .....g............T...........---------------.G................---.T.....---------|............A.........
Caja-F4*04:01      .....g...........................------------..............C.......T..GGTTGGGAAGGG|..G.........A...A.....
Caja-F4*04:03      .....g...........................------------..............C.......T..GGTTGGGAAGGG|..G.........A...A.....
Caja-F4*04:06      .....g...........................------------..............C.......T..GGTTGGGAAGGG|..G.........A...A.....
Caja-F4*04:07      .....g...........................------------..............C.......T..GGTTGGGAAGGG|..G.........A...A.....
Caja-F6*06:01      .....g...........................------------......................T..GGCTGGGAAGGG|..G.........A...A.....
Caja-F6*06:02      .....g...........................------------......................T..GGCTGGGAAGGG|..G.........A...A.....
Caja-F5*04:02N     .....g...........................------------..............C.......T..GGTTGGGAAGGG|..G.....T...A...A.....
Caja-F5*04:04N     .....g...........................------------..............C.......T..GGTTGGGAAGGG|..G.........A...A.....
Caja-F5*04:05N     .....g...........................------------..............C.......T..GGTTGGGAAGGG|..G.....T...A...A.....
Caja-F7*07:01N     .....g.................a.........------------......................T..GGCTGGGAAGGG|............A...A.....
Caja-F1*08:01N     .....g...........................------------......................T.....---------|............A.....G...
Caja-F1*08:02N     .....g...........................------------......................T.....---------|............A.....G...
```

EXON 6   EXON 8

```
                   1030      ||1040
                  ----|----:-||---|-
HLA-F*01:01       TCAGGCTGCAG||TGTGA
HLA-F*01:02       ...........||.....
Patr-F*01:01:02   ...........||.....
Patr-F*01:02:01   ...........||.....
Patr-F*01:03      ...........||.....
Papa-F AF084027   ...........||.....
Gogo-F*01:01      ...........||.....
Gogo-F*01:03      ...........||.....
Poab-F*01:01:01   ..........A||.....
Popy-F*01:01:02   ..........A||.....
Mamu-F*02:01      ....C..A..A||.....
Mamu-F*02:02      ....C..A..A||.....
Mafa-F*02:02:01   ....C..A..A||.....
Mafa-F*02:03:01   ....C..A..A||.....
Mane-F*02:01:01   ....C..A..A||.....
Mane-F*02:02:01   ....C..A..A||.....
Paan-F*02:01:01   ....C..A..A||.....
Paan-F*02:02      ....C..A..A||.....
Ceat-F*02:01      ....C.....A||.....
Ceat-F*02:02      ....C.....A||.....
Saoe-F*03:02      ..........A||.....
Saoe-F*03:03      ..........A||.....
Saoe-F*03:04      ..........A||.....
Aole-F*05:01      ..........A||.....
Aole-F*05:02      ..........A||.....
Aole-F*05:03      ..........A||.....
Aole-F*05:04      ..........A||.....
Aole-F*05:05      ..........A||.....
Caja-F4*04:01     ..........A||.....
Caja-F4*04:03     ..........A||.....
Caja-F4*04:06     ..........A||.....
Caja-F4*04:07     ..........A||.....
Caja-F6*06:01     ..........A||.....
Caja-F6*06:02     ..........A||.....
Caja-F5*04:02N    ..........A||....T
Caja-F5*04:04N    ..........A||.....
Caja-F5*04:05N    ..........A||....T
Caja-F7*07:01N    ..........A||.....
Caja-F1*08:01N    ..........A||.....
Caja-F1*08:02N    ..........A||.....
```
