# Supplementary material for: Evolution of HLA-F and its orthologues in primate species: a complex tale of conservation, diversification and inactivation
Source: Immunogenetics. 2020 Nov 12;72(9):475–87. doi: 10.1007/s00251-020-01187-1 (PMC7725694; doi:10.1007/s00251-020-01187-1)
Supplement: Supplementary file 3 — Supplementary file3: Suppl. Figure 2: DNA alignment of full-length F-like sequences of cynomolgus (Mafa) and rhesus (Mamu) macaques. All allotypes found in this study are included. Lowercase letters refer to synonymous differences as compared with consensus sequence, whereas capitals indicate nonsynonymous changes. (PDF 7.26 MB) [file 251_2020_1187_MOESM3_ESM.pdf]

```
                                              EXON 1                                                  EXON 2

                  10        20        30        40        50        60   |  70        80        90        100       110
         ----:----|----:----|----:----|----:----|----:----|----:----|----|:----|----:----|----:----|----:----|----:----|
Consensus         ATGGCGCCCCGAACCCTCCTCCTGGTGCTCTCAGGGGCCCTGGCCCTGACCGAGACCTGGGCGG|GCTCCCACTCCTTGCGGTATTTTAGCACCGCTGTGTCGCGGCCCGG
Mafa-F*02:02:01   ................................................................|..............................................
Mafa-F*02:03:01   ................................................................|..............................................
Mafa-F*02:04:01   ....a.........................................................a.|..............................................
Mafa-F*02:06:01   ................................................................|..............................................
Mafa-F*02:07      ....a...........................................................|..............................................
Mafa-F*02:08      ................................................................|..............................................
Mafa-F*02:09:01   ................................................................|..............................................
Mafa-F*02:10      ................................................................|..............................................
Mafa-F*02:11      ....a...........................................................|..............................................
Mafa-F*02:12      ................................................................|..............................................
Mafa-F*021:3      ................................................................|..............................................
Mafa-F*02:14      ................................................................|..............................................
Mafa-F*02:15      ................................................................|..............................................
Mafa-F*02:16      ................................................................|..............................................
Mafa-F*02:17      ....a...........................................................|..............................................
Mafa-F*02:18      ....a...........................................................|..............................................
Mafa-F*02:19      ................................................................|..............................................
Mafa-F*02:20      ................................................................|..............................................
Mafa-F*02:21      ................................................................|..............................................
Mafa-F*02:22      ................................................................|..............................................
Mamu-F*02:01      ................................................................|..............................................
Mamu-F*02:02      ................................................................|..............................................
Mamu-F*02:03:01   ................................................................|..............................................
Mamu-F*02:04:01   ................................................................|..............................................
Mamu-F*02:07:01   ................................................................|..............................................
Mamu-F*02:08:01   ....a.................................t.........................|..............................................
Mamu-F*02:09      ................................................................|..............................................
Mamu-F*02:10      ................................................................|..............................................
Mamu-F*02:11      ................................................................|..............................................
Mamu-F*02:12      ................................................................|..............................................
Mamu-F*02:13      ................................................................|..............................................
Mamu-F*02:14      ................................................................|..............................................
Mamu-F*02:15      ................................................................|..............................................
Mamu-F*02:16      ................................................................|..............................................
Mamu-F*02:17      ....a...........................................................|..............................................
Mamu-F*02:18      ................................................................|..............................................
```

EXON 2

```
                       120       130       140       150       160       170       180       190       200       210       220
                ----:----|----:----|----:----|----:----|----:----|----:----|----:----|----:----|----:----|----:----|----:----|
Consensus       CCGCAGGGAGCCTCAGTACAGGTACATCGCGGTGGAGTACGTGGACGACACGCAGTTCCTGCGGTTCGACAGCGACGCCGCGATTCCGAGGATGGAGCCGCGGGCGCCGT
Mafa-F*02:02:01 ......................................................................................................a.......
Mafa-F*02:03:01 ......................................................................................................a.......
Mafa-F*02:04:01 ..............G...............................................................................................
Mafa-F*02:06:01 ..............G...............................................................................................
Mafa-F*02:07    ..............G............................................................................................T..
Mafa-F*02:08    ..............G...............................................................................................
Mafa-F*02:09:01 ......................................................................................................a.......
Mafa-F*02:10    ........................................................................................................A.....
Mafa-F*02:11    ..............................................................................................................
Mafa-F*02:12    ......................................................................................................a.......
Mafa-F*021:3    ......................................................................................................a.......
Mafa-F*02:14    ..............................................................................................................
Mafa-F*02:15    ......................................................................................................a.......
Mafa-F*02:16    ..............G...............................................................................................
Mafa-F*02:17    ..............................................................................................................
Mafa-F*02:18    ..............G...............................................................................................
Mafa-F*02:19    ......................................................................................................a.......
Mafa-F*02:20    ............c.G...............................................................................................
Mafa-F*02:21    ..............G...............................................................................................
Mafa-F*02:22    ..............G...................................................t...........................................
Mamu-F*02:01    ..............G...............................................................................................
Mamu-F*02:02    ......................................................................................................a.......
Mamu-F*02:03:01 ........................................................................................................A.....
Mamu-F*02:04:01 ......................................................................................................a.......
Mamu-F*02:07:01 ......................................................................................................a.......
Mamu-F*02:08:01 ..............G..........................................t....................................................
Mamu-F*02:09    ......................................................................................................a.......
Mamu-F*02:10    .A....................................................................................................a.......
Mamu-F*02:11    ......................................................................................................a.......
Mamu-F*02:12    ........................................................................................................A.....
Mamu-F*02:13    ......................................................................................................a.......
Mamu-F*02:14    .....................................................................................................TA.......
Mamu-F*02:15    ......................................................................................................a.......
Mamu-F*02:16    ..............G...............................................................................................
Mamu-F*02:17    ......................................................................................................a.......
Mamu-F*02:18    ............c.G...............................................................................................
```

EXON 2

```
                       230       240       250       260       270       280       290       300       310       320       330
                ----:----|----:----|----:----|----:----|----:----|----:----|----:----|----:----|----:----|----:----|----:----|
Consensus       GGGTGGAGCAAGAGGGGCCGCAGTATTGGGAGCGGACCACAGGGTACGCCAAGGCCAACGCACGGACTGACCGAGTGGCCCTGAGGAAGCTGCTCCTCCGCTACAACCAG
Mafa-F*02:02:01 ..............................................................................................................
Mafa-F*02:03:01 ..............................................................................................................
Mafa-F*02:04:01 ..............................................................................................................
Mafa-F*02:06:01 ..............................................................................................................
Mafa-F*02:07    ..............................................................................................................
Mafa-F*02:08    ..............................................................................................................
Mafa-F*02:09:01 ..............................................................................................................
Mafa-F*02:10    ..............................................................................................................
Mafa-F*02:11    ..............................................................................................................
Mafa-F*02:12    ..............................................................................................................
Mafa-F*021:3    ..............................................................................................................
Mafa-F*02:14    ..............................................................................................................
Mafa-F*02:15    ..............................................................................................................
Mafa-F*02:16    ..............................................................................................................
Mafa-F*02:17    ..............................................................................................................
Mafa-F*02:18    ..............................................................................................................
Mafa-F*02:19    ..............................................................................................................
Mafa-F*02:20    ..............................................................................................................
Mafa-F*02:21    .................................C............................................................................
Mafa-F*02:22    ..............................................................................................................
Mamu-F*02:01    ..............................................................................................................
Mamu-F*02:02    ..............................................................................................................
Mamu-F*02:03:01 ..............................................................................................................
Mamu-F*02:04:01 ..............................................................................................................
Mamu-F*02:07:01 ..............................................................................................................
Mamu-F*02:08:01 ..............................................................................................................
Mamu-F*02:09    ..............................................................................................................
Mamu-F*02:10    ..............................................................................................................
Mamu-F*02:11    ..............................................................................................................
Mamu-F*02:12    ..............................................................................................................
Mamu-F*02:13    ..............................................................................................................
Mamu-F*02:14    ..............................................................................................................
Mamu-F*02:15    ..............................................................................................................
Mamu-F*02:16    ..............................................................................................................
Mamu-F*02:17    ..............................................................................................................
Mamu-F*02:18    ..............................................................................................................
```

EXON 3

```
                       340|       350       360       370       380       390       400       410       420       430       440
                ----:----||----:----|----:----|----:----|----:----|----:----|----:----|----:----|----:----|----:----|----:----|
Consensus       AGCGAGGCCG|GGTCTCACACCCTCCAGGGAATGAACGGCTGCGACATGGGGCCCGACGGACGCCTCCTCCGCGGGTATCACCAGCACGCCTACGACGGCAAGGATTACAT
Mafa-F*02:02:01 ..........|.........................t..........................................................................
Mafa-F*02:03:01 ..........|....................................................t...............................................
Mafa-F*02:04:01 ..........|...............................................................................t....................
Mafa-F*02:06:01 ..........|....................................................................................................
Mafa-F*02:07    ..........|....................................................................................................
Mafa-F*02:08    ........a.|....................................................................................................
Mafa-F*02:09:01 ..........|....................................................................................................
Mafa-F*02:10    ........a.|....................................................................................................
Mafa-F*02:11    ........a.|.........................t..........................t...............................................
Mafa-F*02:12    ..........|.........................t..........................................................................
Mafa-F*021:3    ..........|....................................................................................................
Mafa-F*02:14    ........a.|.........................t..........................t..............................A................
Mafa-F*02:15    ..........|....................................................................................................
Mafa-F*02:16    ........a.|....................................................................................................
Mafa-F*02:17    ..........|....................................................................................................
Mafa-F*02:18    ..........|...............................................................................t....................
Mafa-F*02:19    ..........|.........................t......................A...................................................
Mafa-F*02:20    ........a.|....................................................................................................
Mafa-F*02:21    ..........|....................................................................................................
Mafa-F*02:22    ........a.|....................................................................................................
Mamu-F*02:01    ........a.|....................................................................................................
Mamu-F*02:02    ..........|....................................................................................................
Mamu-F*02:03:01 ........a.|....................................................................................................
Mamu-F*02:04:01 ..........|....................................................................................................
Mamu-F*02:07:01 ..........|....................................................t...............................................
Mamu-F*02:08:01 ..........|....................................................................................................
Mamu-F*02:09    ..........|....................................................................................................
Mamu-F*02:10    ..........|....................................................t...............................................
Mamu-F*02:11    ..........|....................................................t...............................................
Mamu-F*02:12    ........a.|....................................................................................................
Mamu-F*02:13    ..........|..................................................................................................G.
Mamu-F*02:14    ..........|....................................................................................................
Mamu-F*02:15    ..........|.........................t..........................................................................
Mamu-F*02:16    ..........|....................................................................................................
Mamu-F*02:17    ..........|.........................t..........................................................................
Mamu-F*02:18    ........a.|....................................................................................................
```

EXON 3

```
                       450       460       470       480       490       500       510       520       530       540       550
                ----:----|----:----|----:----|----:----|----:----|----:----|----:----|----:----|----:----|----:----|----:----|
Consensus       CTCCCTGAACGAGGACCTGCGCTCCTGGACCGCCGCGGACACGGTAGCTCGGATCACCCAGCGCTTCTATGAGGCAGAGGAATATGCAGAGGAGTTCAGGACCTACCTGG
Mafa-F*02:02:01 ..............................................................................................................
Mafa-F*02:03:01 ..............................................................................................................
Mafa-F*02:04:01 ..............................................................................................................
Mafa-F*02:06:01 ..............................................................................................................
Mafa-F*02:07    ..............................................................................................................
Mafa-F*02:08    ..............................................................................................................
Mafa-F*02:09:01 ..............................................................................................................
Mafa-F*02:10    ..............................................................................................................
Mafa-F*02:11    ..............................................................................................................
Mafa-F*02:12    ..............................................................................................................
Mafa-F*021:3    ..............................................................................................................
Mafa-F*02:14    ..............................................................................................................
Mafa-F*02:15    ..............................................................................................................
Mafa-F*02:16    ..............................................................................................................
Mafa-F*02:17    ..............................................................................................................
Mafa-F*02:18    ..............................................................................................................
Mafa-F*02:19    ..............................................................................................................
Mafa-F*02:20    ..............................................................................................................
Mafa-F*02:21    ..............................................................................................................
Mafa-F*02:22    ..............................................................................................................
Mamu-F*02:01    .....................................................................................................T........
Mamu-F*02:02    ..............................................................................................................
Mamu-F*02:03:01 ..............................................................................................................
Mamu-F*02:04:01 ..............................................................................................................
Mamu-F*02:07:01 ..............................................................................................................
Mamu-F*02:08:01 ..............................................................................................................
Mamu-F*02:09    ..............................................................................................................
Mamu-F*02:10    ..............................................................................................................
Mamu-F*02:11    ..............................................................................................................
Mamu-F*02:12    ..............................................................................................................
Mamu-F*02:13    ..............................................................................................................
Mamu-F*02:14    ..............................................................................................................
Mamu-F*02:15    ..............................................................................................................
Mamu-F*02:16    ..............................................................................................................
Mamu-F*02:17    ..............................................................................................................
Mamu-F*02:18    ..............................................................................................................
```

```
                                  EXON 3                                                EXON 4

                         560       570       580       590       600       610      | 620       630       640       650       660
                ----:----|----:----|----:----|----:----|----:----|----:----|------|-|----:----|----:----|----:----|----:----|
Consensus       AGGGCGAGTGCCTGGAGTTGCTCCGCAGATACCTGGAGAACGGGAAGGAGACGCTACAGCGCGCAG|ATCCTCCAAAGGCACACGTTGCCCACCACCCCATCTCTGACCGT
Mafa-F*02:02:01 ..................................................................|............................................
Mafa-F*02:03:01 ..................................................................|............................................
Mafa-F*02:04:01 ..................................................................|..............................A.............
Mafa-F*02:06:01 ..................................................................|............................................
Mafa-F*02:07    ..................................................................|..............................A.............
Mafa-F*02:08    ..................................................................|............................................
Mafa-F*02:09:01 ..................................................................|............................................
Mafa-F*02:10    ..................................................................|............................................
Mafa-F*02:11    ..................................................................|..............................A.............
Mafa-F*02:12    ..................................................................|..........................................A.
Mafa-F*021:3    ..................................................................|............................................
Mafa-F*02:14    ..................................................................|..............................A.............
Mafa-F*02:15    ..................................................................|............................................
Mafa-F*02:16    ..............................................................C...|............................................
Mafa-F*02:17    ..................................................................|..............................A.............
Mafa-F*02:18    ..................................................................|..............................A.............
Mafa-F*02:19    ..................................................................|..............................A.............
Mafa-F*02:20    ..................................................................|............................................
Mafa-F*02:21    ..................................................................|............................................
Mafa-F*02:22    ..................................................................|............................................
Mamu-F*02:01    ..................................................................|............................................
Mamu-F*02:02    ..................................................................|............................................
Mamu-F*02:03:01 ..................................................................|............................................
Mamu-F*02:04:01 ..................................................................|............................................
Mamu-F*02:07:01 ..................................................................|............................................
Mamu-F*02:08:01 ..................................................................|............................................
Mamu-F*02:09    ..................................................................|............................................
Mamu-F*02:10    ..................................................................|............................................
Mamu-F*02:11    ..................................................................|............................................
Mamu-F*02:12    ..................................................................|............................................
Mamu-F*02:13    ..................................................................|............................................
Mamu-F*02:14    ..................................................................|............................................
Mamu-F*02:15    ..................................................................|............................................
Mamu-F*02:16    ..................................................................|............................................
Mamu-F*02:17    ........................C.........................................|............................................
Mamu-F*02:18    ..................................................................|............................................
```

EXON 4

```
                  670       680       690       700       710       720       730       740       750       760       770
             ----:----|----:----|----:----|----:----|----:----|----:----|----:----|----:----|----:----|----:----|----:----|
Consensus    GAGGCCACCCTGAGGTGCTGGGCCCTGGGCTTCTACCCTGCCGAGATCACACTGACCTGGCAGCGGGACGGGGAGGAACAGACCCAGGACACGGAGCTTGTGGAGACCAG
Mafa-F*02:02:01 ..............................................................................................................
Mafa-F*02:03:01 ..............................................................................................................
Mafa-F*02:04:01 ..............................................................................................................
Mafa-F*02:06:01 ..............................................................................................................
Mafa-F*02:07 ..............................................................................................................
Mafa-F*02:08 ..............................................................................................................
Mafa-F*02:09:01 ....................................................................t.........................................
Mafa-F*02:10 ..............................................................................................................
Mafa-F*02:11 ..............................................................................................................
Mafa-F*02:12 ..............................................................................................................
Mafa-F*021:3 ....................................................................A.........................................
Mafa-F*02:14 ..............................................................................................................
Mafa-F*02:15 ..........................................A...................................................................
Mafa-F*02:16 ..............................................................................................................
Mafa-F*02:17 ..............................................................................................................
Mafa-F*02:18 ..............................................................................................................
Mafa-F*02:19 ..............................................................................................................
Mafa-F*02:20 ..............................................................................................................
Mafa-F*02:21 ..............................................................................................................
Mafa-F*02:22 ...........................................................................................T..................
Mamu-F*02:01 ..............................................................................................................
Mamu-F*02:02 ..............................................................................................................
Mamu-F*02:03:01 ..............................................................................................................
Mamu-F*02:04:01 ..............................................................................................................
Mamu-F*02:07:01 ..............................................................................................................
Mamu-F*02:08:01 ..............................................................................................................
Mamu-F*02:09 ....................................................................t.........................................
Mamu-F*02:10 .........................................t.......................................C.g..........................
Mamu-F*02:11 .................................................................................C............................
Mamu-F*02:12 ..............................................................................................................
Mamu-F*02:13 ..............................................................................................................
Mamu-F*02:14 ....................................................................t.........................................
Mamu-F*02:15 ..............................................................................................................
Mamu-F*02:16 .........................................g....................................................................
Mamu-F*02:17 ..............................................................................................................
Mamu-F*02:18 ..............................................................................................................
```

EXON 4

```
                       780       790       800       810       820       830       840       850       860       870       880
                ----:----|----:----|----:----|----:----|----:----|----:----|----:----|----:----|----:----|----:----|----:----|
Consensus       GCCTGCAGGGGATGGAACCTTCCAGAAGTGGGCAGCTGTGGTGGTGCCTTCCGGAGAGGAGCAGAGATACACGTGCCATGTGCAACACGAAGGGCTGCCCCAGCCCCTCA
Mafa-F*02:02:01 ..............................................................................................................
Mafa-F*02:03:01 ..............................................................................................................
Mafa-F*02:04:01 .....................................................................t........................................
Mafa-F*02:06:01 ..............................................................................................................
Mafa-F*02:07    ..............................................................................................................
Mafa-F*02:08    ..............................................................................................................
Mafa-F*02:09:01 ..............................................................................................................
Mafa-F*02:10    ..............................................................................................................
Mafa-F*02:11    .................................g............................................................................
Mafa-F*02:12    ..............................................................................................................
Mafa-F*021:3    ..............................................................................................................
Mafa-F*02:14    ..............................................................................................................
Mafa-F*02:15    ..............................................................................................................
Mafa-F*02:16    ..............................................................................................................
Mafa-F*02:17    .................................g................................................................T...........
Mafa-F*02:18    .......................................................................T......................................
Mafa-F*02:19    ..............................................................................................................
Mafa-F*02:20    ...............................................T..............................................................
Mafa-F*02:21    ..............................................................................................................
Mafa-F*02:22    ..............................................................................................................
Mamu-F*02:01    ..............................................................................................................
Mamu-F*02:02    ..................................................................................................T...........
Mamu-F*02:03:01 ..............................................................................................................
Mamu-F*02:04:01 ..............................................................................................................
Mamu-F*02:07:01 ..............................................................................................................
Mamu-F*02:08:01 ..............................................................................................................
Mamu-F*02:09    ..............................................................................................................
Mamu-F*02:10    ..............................................................................................................
Mamu-F*02:11    ..............................................................................................................
Mamu-F*02:12    ..............................................................................................................
Mamu-F*02:13    ..............................................................................................................
Mamu-F*02:14    ..............................................................................................................
Mamu-F*02:15    ..............C...........................................................................g...................
Mamu-F*02:16    ..............................................................................................................
Mamu-F*02:17    ..............................................................................................................
Mamu-F*02:18    ...............................................T..............................................................
```

EXON 5

```
                 890 |     900       910       920       930       940       950       960       970       980       990
                 ----:----|--|--:----|----:----|----:----|----:----|----:----|----:----|----:----|----:----|----:----|----:----|
Consensus        CCCTGAGATGGG|AGTCGTCTTCTCAGCCCACCATCCCCATTGTGGGCATCGTTGCTGGCCTGGCTGTCCTAGCAGTTGTGGTTACTGGAGCTGTGGTCGCTGCTGTAATG
Mafa-F*02:02:01  ............|..................................................................................................
Mafa-F*02:03:01  ............|......................................................................................A...........
Mafa-F*02:04:01  ............|......................................................................c...........................
Mafa-F*02:06:01  ............|......................................................................c...........................
Mafa-F*02:07     ............|......................................................................c...........................
Mafa-F*02:08     ............|..................................................................................................
Mafa-F*02:09:01  ............|......................................................................c...........................
Mafa-F*02:10     ............|..................................................................................................
Mafa-F*02:11     ............|..................................................................................................
Mafa-F*02:12     ............|..................................................c...................................A...........
Mafa-F*021:3     ............|......................................................................c...........................
Mafa-F*02:14     ............|......................................................................c...........................
Mafa-F*02:15     ............|......................................................................................A...........
Mafa-F*02:16     ............|......................................................................................A...........
Mafa-F*02:17     ............|..................................................................................................
Mafa-F*02:18     ............|......................................................................c...........................
Mafa-F*02:19     ............|......................................................................c...........................
Mafa-F*02:20     ............|......................................................................................A...........
Mafa-F*02:21     ............|......................................................................c...........................
Mafa-F*02:22     ............|..................................................................................................
Mamu-F*02:01     ............|..................................................................................................
Mamu-F*02:02     ............|..................................................................................................
Mamu-F*02:03:01  ............|......................................................................................A...........
Mamu-F*02:04:01  ............|..................................................................................................
Mamu-F*02:07:01  ............|......................................................................................A...........
Mamu-F*02:08:01  ............|..................................................................................................
Mamu-F*02:09     ............|......................................................................c...........................
Mamu-F*02:10     ............|......................................................................................A...........
Mamu-F*02:11     ............|......................................................................................A...........
Mamu-F*02:12     ............|..................................................................................................
Mamu-F*02:13     ............|......................................................................................A...........
Mamu-F*02:14     ............|......................................................................................A...........
Mamu-F*02:15     ............|..................................................................................................
Mamu-F*02:16     ............|......................................................................................A...........
Mamu-F*02:17     ............|..................................................................................................
Mamu-F*02:18     ............|......................................................................................A...........
```

```
                                       EXON 6             EXON 8

                  1000      101|0      1020      1030      1040
                ----:----|----:----||----:----|----:----|----:----|--||--:--
Consensus       TGGAGGAGAAAGAGCTCAG|ATAGAAACAGAGGGAGCTACTCTCAGCCTACAA||TGTGA
Mafa-F*02:02:01 ...................|.................................||.....
Mafa-F*02:03:01 ...................|.................................||.....
Mafa-F*02:04:01 ....A...g..........|.................................||.....
Mafa-F*02:06:01 ....A...g..........|.................................||.....
Mafa-F*02:07    ....A...g..........|.................................||.....
Mafa-F*02:08    ...................|.................................||.....
Mafa-F*02:09:01 ....A...g..........|.................................||.....
Mafa-F*02:10    ...................|.................................||.....
Mafa-F*02:11    ...................|.................................||.....
Mafa-F*02:12    ...................|.................................||.....
Mafa-F*021:3    ....A...g..........|.................................||.....
Mafa-F*02:14    ....A...g..........|.................................||.....
Mafa-F*02:15    ...................|.................................||.....
Mafa-F*02:16    ...................|.................................||.....
Mafa-F*02:17    ...................|.................................||.....
Mafa-F*02:18    ....A...g..........|.................................||.....
Mafa-F*02:19    ....A...g..........|.................................||.....
Mafa-F*02:20    ...................|.................................||.....
Mafa-F*02:21    ...................|.................................||.....
Mafa-F*02:22    ...................|.................................||.....
Mamu-F*02:01    ...................|.................................||.....
Mamu-F*02:02    ...................|.................................||.....
Mamu-F*02:03:01 ...................|.................................||.....
Mamu-F*02:04:01 ...................|.................................||.....
Mamu-F*02:07:01 ...................|.................................||.....
Mamu-F*02:08:01 ...................|.................................||.....
Mamu-F*02:09    ....A...g..........|.................................||.....
Mamu-F*02:10    ...................|.................................||.....
Mamu-F*02:11    ...................|.................................||.....
Mamu-F*02:12    ...................|.................................||.....
Mamu-F*02:13    ...................|.................................||.....
Mamu-F*02:14    ...................|.................................||.....
Mamu-F*02:15    ...................|.................................||.....
Mamu-F*02:16    ...................|.................................||.....
Mamu-F*02:17    ...................|.................................||.....
Mamu-F*02:18    ...................|.................................||.....
```
